# Essential Roles of the Linker Sequence Between Tetratricopeptide Repeat Motifs of Ethylene Overproduction 1 in Ethylene Biosynthesis

**DOI:** 10.3389/fpls.2021.657300

**Published:** 2021-04-15

**Authors:** Chuanjing An, Yuefang Gao

**Affiliations:** ^1^State Key Laboratory of Natural and Biomimetic Drugs, Department of Chemical Biology, School of Pharmaceutical Sciences, Peking University, Beijing, China; ^2^College of Horticulture, Northwest A&F University, Yangling, China

**Keywords:** ACSs, ethylene, ethylene biosynthesis, ethylene overproduction 1, tetratricopeptide repeat, triple response

## Abstract

Ethylene Overproduction 1 (ETO1) is a negative regulator of ethylene biosynthesis. However, the regulation mechanism of ETO1 remains largely unclear. Here, a novel *eto1* allele (*eto1-16*) was isolated with typical triple phenotypes due to an amino acid substitution of G480C in the uncharacterized linker sequence between the TPR1 and TPR2 motifs. Further genetic and biochemical experiments confirmed the *eto1-16* mutation site. Sequence analysis revealed that G480 is conserved not only in two paralogs, EOL1 and EOL2, in *Arabidopsis*, but also in the homologous protein in other species. The glycine mutations (*eto1-11*, *eto1-12*, and *eto1-16*) do not influence the mRNA abundance of *ETO1*, which is reflected by the mRNA secondary structure similar to that of WT. According to the protein-protein interaction analysis, the abnormal root phenotype of *eto1-16* might be caused by the disruption of the interaction with type 2 1-aminocyclopropane-1-carboxylic acid (ACC) synthases (ACSs) proteins. Overall, these data suggest that the linker sequence between tetratricopeptide repeat (TPR) motifs and the glycine in TPR motifs or the linker region are essential for ETO1 to bind with downstream mediators, which strengthens our knowledge of ETO1 regulation in balancing ACSs.

## Introduction

Ethylene, a simple gaseous plant hormone, is involved in numerous aspects of plant growth and development processes, including seed germination and shoot and root growth and development (Lin et al., 2009; Ahammed et al., 2020; Binder, 2020). Most plant cells can produce ethylene, a process which increases during certain developmental stages, such as fruit ripening, senescence, and leaf abscission (Liu et al., 2015; Dubois et al., 2018; Li et al., 2019) and responds to biotic and abiotic cues, such as flooding, heavy metals, heat, drought, ozone, phosphate starvation, and soil alkalinity (Lei et al., 2011; Habben et al., 2014; Steffens, 2014; Pan et al., 2019; Wang et al., 2019; Huang and Zhang, 2020; Perata, 2020; Hartman et al., 2021). The ethylene biosynthesis pathway in higher plants is well elucidated (Ecker, 1995; Lin et al., 2009; Bakshi et al., 2015; Binder, 2020). Ethylene originates from methionine through a series of biochemical reactions. Firstly, methionine adenosyltransferase catalyzes the conversion of methionine into S-adenosylmethionine (SAM; Yang and Hoffman, 1984). Subsequently, SAM is converted into 1-aminocyclopropane-1-carboxylic acid (ACC) by ACC synthase (ACS). Finally, ACC is transformed into ethylene by ACC oxidase (ACO; Rodrigues et al., 2014; Bakshi et al., 2015; Binder, 2020).

In *Arabidopsis*, ethylene is recognized by receptors on the endoplasmic reticulum membrane, including ethylene response 1 (ETR1), ETR2, ethylene response sensor 1 (ERS1), ERS2, and ethylene insensitive 4 (EIN4; Grefen et al., 2008; Ju and Chang, 2015). Constitutive triple response 1 (CTR1), a serine/threonine Raf-like kinase, is a negative regulator in ethylene signaling as it can bind to the receptors (Mayerhofer et al., 2012; Yasumura et al., 2015). The activation (receptor bound form) and inactivation (receptor released form) of CTR1 is controlled by the absence or presence of ethylene, respectively, which further switches the phosphorylation and non-phosphorylation states of the positive regulator EIN2 (Zhao et al., 2021). In the absence of ethylene, CTR1 activation leads to the phosphorylation of the C-terminal domain of EIN2, which targets the EIN2 for degradation, leading to the repression of ethylene responses in plants. In contrast, the cytosolic C-terminus of non-phosphorylated EIN2 is released and targeted to the nucleus, which promotes the stability of EIN3 and EIN3 LIKE 1 (EIL1) transcription factors (Alonso et al., 1999; Ju et al., 2012; Zhang et al., 2017). Consequently, ethylene induces a rapid accumulation of EIN3/EIL to promote transcriptional cascades of ethylene response genes (Potuschak et al., 2003; Li et al., 2015; Merchante et al., 2015; Dolgikh et al., 2019). Ethylene biosynthesis, perception, and signal transduction pathways regulate ethylene responses integrally (Merchante et al., 2013; Binder, 2020).

In ethylene biosynthesis, the conversion of SAM to ACC, which is catalyzed by ACSs, is a rate-limiting step. ACSs are encoded by a multigene family in plants, which are classified into three subgroups based on their protein sequences and domain structures. In the *Arabidopsis* genome, there are eight active *ACS*s (type 1: ACS2, type 2: ACS4-9, and type 3: ACS11) and an inactive *ACS1* (Liang et al., 1995; Yamagami et al., 2003; Tsuchisaka et al., 2009), all of which can participate in ethylene production. The elimination of the entire gene family results in embryonic lethality; however, an *acs* octuple-mutant is able to survive (Tsuchisaka et al., 2009). The nine genes are differentially expressed in response to various developmental, environmental, and hormonal factors. Among them, *ACS4* is responsive to auxin (Abel et al., 1995), *ACS5* to cytokinin (Vogel et al., 1998), and *ACS6* to ozone and other stimuli (Chen et al., 2020). In addition, at the post-transcriptional level, ACS quantities and activities are under rigorous and dynamic regulation, including ubiquitin-26S proteasome degradation (Wang et al., 2004; Lyzenga et al., 2012), proteolysis, and reversible phosphorylation by various protein kinases and phosphatases (Spanu et al., 1994; Liu and Zhang, 2004; Kamiyoshihara et al., 2010; Skottke et al., 2011; Seo and Yoon, 2019).

In *Arabidopsis*, ethylene overproduction 1 (ETO1) is a negative regulator *via* the regulation of ACS stability (Woeste et al., 1999; Wang et al., 2004; Yoshida et al., 2006). ETO1 encodes a protein containing a Broad-complex, Tramtrack, Bric-a-brac (BTB) domain functioning in E3 ligase interactions, and seven tetratricopeptide repeat (TPR) motifs, which can directly bind to the C-terminal extension of ACS5 (Wang et al., 2004). Upon such binding, ETO1 inhibits the enzymatic activity and protein stability of ACS5 through interacting with CUL3 proteins, which can target ACS5 for degradation mediated by 26S proteasome (Chae et al., 2003; Wang et al., 2004; Yoshida et al., 2005). To date, numerous studies have investigated on *ETO1* function, and nearly 20 *eto1* mutants have been isolated (Wang et al., 2004; Ortega-Martinez et al., 2007). Mutation of ETO1 leads to ACS5 protein accumulation, higher ethylene production, and the typical triple responses in *Arabidopsis* seedlings (Chae et al., 2003). In contrast, ETO1 overexpression lines decrease the ACS5 level and ethylene production (Wang et al., 2004). As a regulatory hub in ethylene biosynthesis, numerous accessory proteins that bind to ETO1 or ACSs add layers of complexity to the regulation of the activity and abundance of ACSs; for example, protein kinase CK1.8 regulates the interaction between ACS5 and ETO1 (Tan and Xue, 2014), and 14-3-3 is involved in ACS5 stabilization and ETO1 destabilization (Yoon and Kieber, 2013). However, to date, the function and mechanism underlying ETO1 regulation remain largely unclear, especially in relation to the uncharacterized long linker sequence between TPR motifs.

Here, we reported a mutant, *eto1-16*, with an altered root phenotype, which possesses an amino acid substitution of glycine to cysteine in the linker sequence between TPR1 and TPR2 motifs of ETO1. Further biochemical analyses revealed that the *eto1-16* site mutation disrupts the interaction of ETO1 with the ACS5 C-terminus but does not affect the interaction with CUL3A, which results in the accumulation of ACS proteins, and ultimately high ethylene levels. According to our results, the large linker region between TPR motifs is essential for ETO1 in protein-protein interactions. Substitution of the small-sized glycine by amino acids with large side chains might inhibit conformation alteration, which is important for protein-protein interaction. The multilayer regulation of proteins ETO1 and ACSs, through mechanisms such as phosphorylation of ACSs by CK1.8 and stability control by 14-3-3, facilitates ethylene production within a balanced range and rapid response to internal or external cues. This study provides further insights into the role of ETO1 in the regulation of ethylene biosynthesis, including ACS activity and stability.

## Materials and Methods

### Plants Materials

Wild-type (WT), homozygous mutants, and transgenic *Arabidopsis thaliana* (Columbia) seeds were surface sterilized in 75% alcohol and dispersed on Petri dishes containing Murashige and Skoog (MS) salts. Plates were incubated at 4°C for 2 days to promote and synchronize germination and then grown vertically in an incubator at 21 ± 1°C under 16 h illumination or darkness for the observation of root and hypocotyl phenotypes, respectively. For the AgNO_3_ (10 μM) and MG132 (50 μM) treatments, used for inhibiting the ethylene perception and the 26S proteasome protein degradation respectively, appropriate dilutions were prepared from stock solutions for inclusion in the media. Homozygous mutants (*eto1-16eto1-1*, *eto1-16ctr1*, *eto1-16ein2*, and *eto1-1eto2*) were generated by genetic crossing using standard techniques, and the mutant seedlings were confirmed by PCR-based genotyping. The *Arabidopsis* for the complementary assay and transcriptional analysis, and the *Nicotiana benthamiana* for the bimolecular fluorescence complementation (BiFC) assay were grown in soil under conditions similar to those described above.

### Map-Based Cloning of *eto1-16*

*eto1-16* was obtained by chemical mutagenesis with 0.5% ethyl methanesulfonate for 12 h. The mutation site of *eto1-16* was confirmed by a standard map-based cloning protocols as previously described (Hou et al., 2010). The final mapping interval is between CH3 19.20–19.35, in which *ETO1* located.

### DNA Amplification and Sequencing, and Transgenic Protocols

The full length *ETO1* genomic sequence of WT and *eto1-16* was amplified by I-5™ 2 × High-Fidelity Master Mix (MCLAB, South San Francisco, CA, United States) using primers *ETO1-1* and *ETO1-2* for the complementary experiment and mutation site sequencing. The WT version *ETO1*, *MYC-ACS5*, *HA-ETO1*, and *HA-ETO1^G480C^* were cloned into the 3302Y3 vector under their native promoters, which are 1 kb upstream of the start codon for both *ACS5* and *ETO1*. The MYC-tag and HA-tag were fused to the N-terminus of the protein using the PCR technique. In the T3 plant construction, the homozygous mutant *eto1-1eto2* was transformed concurrently with plasmids containing *MYC-ACS5* and *HA-ETO1* fusions. For 14-3-3 RNAi plants, interference DNA segment (genomic ETO1^240–680^ fusion with the inverted DNA^240–449^), including the first intron of *14-3-3ω*, was amplified and ligated into the 1391Z vector. After sequencing, the plasmids were transferred into *Agrobacterium* strain C58C1 and then transformed into T3 plants using the floral dipping method. The transgenic plants were selected using glufosinate, and hygromycin B. Primer sequences are listed in Supplementary Table S1.

### Sequence Alignment and Phylogenetic Analysis

To confirm the mutation site in *eto1-16*, the sequencing results of *eto1-16* were aligned in the Araport11 database.^1^ For the conservation analysis, the protein sequences of ETO1, EOL1, and EOL2 in *Arabidopsis*, and ETO1 homologs in other species were retrieved from the NCBI database.^2^ Multiple alignment was performed using Clustal X (Sievers et al., 2011), and evolutionary analyses were performed in MEGA X (Kumar et al., 2018). The evolutionary history was inferred using the Neighbor-Joining method. The percentages of replicate trees in which the associated taxa clustered together in the bootstrap test (100 replicates) are shown next to the branches.

### Measurement of Ethylene Biosynthesis

Ethylene production measurement was performed as described previously (Christians et al., 2009). Seeds of WT, *eto1-16*, complementary lines (*ETO1*/*ETO1*) and *eto1-16* treated with AgNO_3_ were surface-sterilized, germinated, and grown in 22 ml gas chromatography (GC) vials containing 3 ml of full-strength solid MS medium plus 1% sucrose. The vials were capped and incubated at 22°C for 4 days in the dark, and then frozen at −20°C; the accumulated ethylene was measured by GC. Each of three replicate samples were measured at least three times. Ethylene production was calculated as pL·seedling^−1^·day^−1^.

### Reverse Transcription PCR and Quantitative Real-Time PCR

Total RNA was extracted from WT and *eto1-16* using a PLANTPure Plant RNA Kit (Aidlab, Beijing, China). Total RNA (1 μg) was used for the first strand cDNA synthesis using a RevertAid™ cDNA Synthesis Kit (Thermo Fisher Scientific, Waltham, MA, United States). *ETO1* transcription level was analyzed by reverse transcription PCR (RT-PCR) analysis of total RNA with specific primers (Supplementary Table S1). Two fragments of *ETO1* cDNA, *ETO1-a* (from 1,258 to 2036 bp), and *ETO1-b* (from 2,308 to 2,641 bp) were amplified by RT-PCR, while *Actin7* was used as the control. The PCR products were analyzed by agarose gel electrophoresis.

Quantitative real-time PCR (qRT-PCR) assays were performed with the cDNA of the WT and *eto1-16* plants with specific primers (Supplementary Table S1) using the Hieff® qPCR SYBR Green Master Mix (Low Rox Plus; Yeasen, Shanghai, China), according to the manufacturer’s protocol. Fluorescence emitted from SYBR green was detected using an ABI QuantStudio 6 Real-Time PCR System (Applied Biosystems, Foster City, CA, United States). PCR reactions were performed in quadruplicate for each sample, and the relative expression level for each gene was calculated using the Delta-Delta cycle threshold (Ct) method, where *Actin7* acted as a reference gene.

### Bioinformatics

RNA secondary structure was predicted by RNAfold 2.4.13.^3^ Helix structure analysis was performed with HeliQuest webpage tool.^4^ Structure similarity search was performed with SWISS-MODEL.^5^

### Yeast Two Hybrid Assay and Yeast Three Hybrid Assay

For the yeast two hybrid (Y2H) assay, coding sequences of *Arabidopsis* type 2 ACSs (ACS4, ACS5, and ACS9), 14-3-3ω and CUL3A were amplified and cloned into pGADT7 prey plasmids, and ETO1 into pGBKT7 bait plasmids. The candidate interaction pairs were co-transformed into the AH109 yeast strain (Clontech, United States). The transformed yeast cells were selected on synthetic dropout (-Leu/-Trp) medium. For auxotroph assays, clones were streaked on synthetic dropout (-Leu/-Trp/-His) medium and grown at 30°C for 4 days. Continuous growth in colonies indicated interactions. Each experiment was repeated at least three times using independent clones.

To detect the linker role of ETO1 in ACS5 interaction with CUL3A using the yeast three hybrid (Y3H) assay, a coding sequence of ACS5 was cloned into the pGADT7 prey plasmid, while CUL3A and ETO1 were cloned into the multiple cloning site (MCS) I and II regions of the pBridge plasmid, respectively. To detect the regulatory role of 14-3-3ω in the stability of ETO1 and ACS5, the binding affinity of ETO1 with CUL3A and ACS5 was examined with or without 14-3-3ω. CUL3A and ACS5 were fused to the binding domain before the MCS I sequence, while 14-3-3ω was cloned into the MCS II region of the pBridge plasmid. ETO1 was cloned into the pGADT7 prey plasmid. The candidate interaction pairs were co-transformed into the AH109 yeast strain, and the transformed yeast clones were selected on synthetic dropout (-Leu/-Trp/-Met) medium. For auxotroph assays, four individual clones were streaked on synthetic dropout (-Leu/-Trp/-His/-Met) medium, and grown at 30°C for 4 days.

The primers used are listed in Supplementary Table S1.

### Bimolecular Fluorescence Complementation Assay

For the BiFC assay, coding sequences of ACS4, ACS5, ACS9, and CUL3A were amplified from cDNA and fused with cYFP in 3302YC, and ETO1 with nYFP in 3302YN. Subsequently, the constructs were transformed into *Agrobacterium* strain C58C1 as described above. *Agrobacterium tumefaciens*-mediated transient expression in *N. benthamiana* leaves was performed as previously described (An et al., 2017). Before infiltration, the pairs (ACSs in 3302YC and ETO1 in 3302YN; CUL3A in 3302YC and ETO1 in 3302YN) were mixed evenly with equal doses (OD_600_ = 0.2 for each sample). Leaves of 4-week-old *N. benthamiana* plants were infiltrated using a needleless syringe with a suspension of *Agrobacterium* and incubated for 48 h at 22°C.

### Microscopy and Morphometric Analysis

Image of root and hypocotyl phenotypes were obtained using a camera or under a dissecting microscope. Root and hypocotyl lengths in different plants were measured using a millimeter ruler. Root density and root tip lengths were compared using an ocular micrometer at the same magnification. The fluorescence in tobacco leaves containing BiFC-YFP constructs was observed under a conventional fluorescence microscope (Olympus BX61) and captured with a CCD camera.

### Western Blot Analysis

After growth for 5 days on MS medium, approximately 10 seedlings, homozygous for both transgenes (MYC-ACS5 and HA-ETO1), were collected and rapidly frozen in liquid nitrogen. Total protein was isolated by homogenization in 50 μl of SDS sample buffer (125 mM Tris-HCl, pH 6.8, 4% SDS, 20% glycerol, and 10% 2-mercaptoethanol), and was then clarified by centrifugation (10,000 *g*) at 4°C. The extracts were subjected to SDS-PAGE and immunoblot analysis using anti-MYC and anti-HA antibodies. Coomassie Brilliant Blue staining was used as a loading control.

### Statistical Analysis

GraphPad Prism 7.0 was used to perform statistical analysis. Details of the analysis are mentioned in the respective Figure legend.

## Results

### Typical Triple Response Phenotype of *eto1-16* Mutant

**Figure 1:**
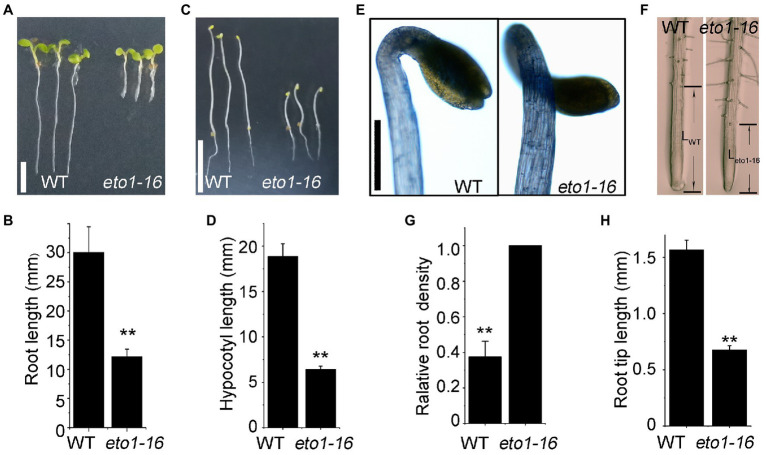
Root and hypocotyl phenotypes of *eto1-16*. **(A)** Root phenotypes of wild-type (WT) and *eto1-16* grown under a 16/8 h light/dark period. Scale bar = 10 mm. **(B)** Statistical analysis of the root length of plants in **(A)**. **(C)** Hypocotyl phenotypes of WT and *eto1-16* grown in the dark. Scale bar = 10 mm. **(D)** Statistical analysis of the hypocotyl length of plants in **(B)**. **(E)** The apical hook phenotype of *eto1-16*. Scale bar = 1 mm. **(F)** Root tip phenotypes of WT and *eto1-16*. The relative root density **(G)** and the root tip length **(H)** of plants in **(D)**. Statistical analysis was performed using a two-tailed Student’s *t*-test (^**^p < 0.01). At least 50 plants were used in root and hypocotyl phenotype tests.

In an EMS mutagenesis library, an individual plant with root phenotype defects attracted our interests, which is a novel *eto1* allele, named *eto1-16*. The root length of 5-day-old *eto1-16* plants grown in MS medium was approximately one-third of the root length of WT plants (Figures 1A,B). The hypocotyl phenotype of etiolated *eto1-16* seedlings grown in the dark was also shorter in length, but wider in diameter, in comparison with that of WT plants (Figures 1C–E). In contrast, root hairs of *eto1-16* were relatively denser and longer than those of the WT. Further microscopic analyses also revealed increased root hair density (Figures 1F,G) and extremely short elongation zones in the *eto1-16* roots in comparison with those of WT plants (Figures 1F,H). The growth rate of *eto1-16* plants was marginally lower than that of the WT plants, with no considerable differences in the case of adult plants (Supplementary Figure S1).

### *eto1-16* Is a Novel *eto1* Allele With an Amino Acid Substitution of Glycine to Cysteine

**Figure 2:**
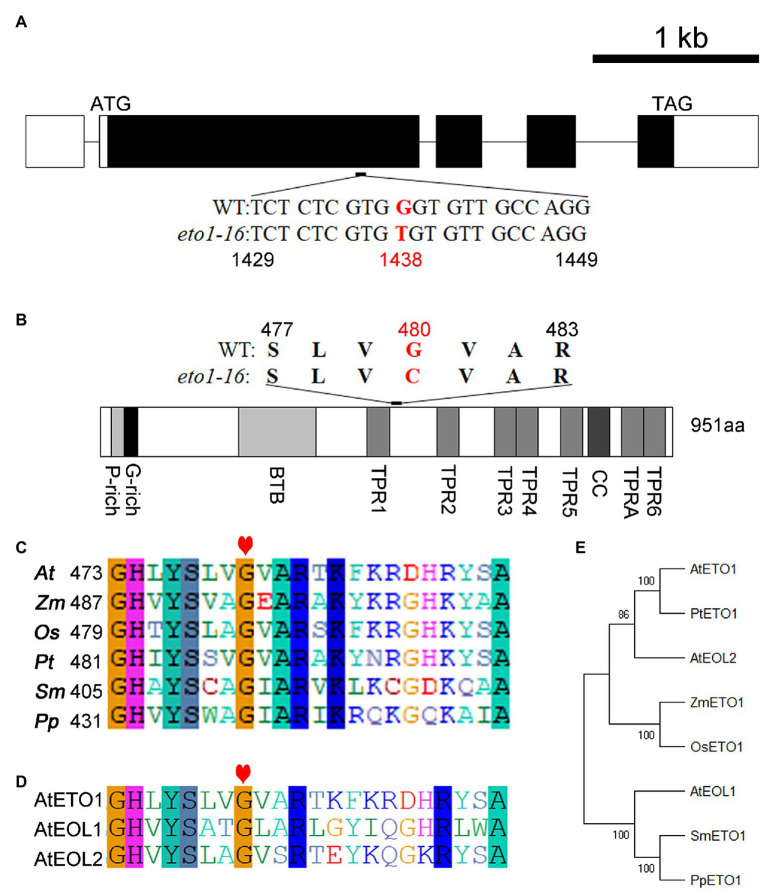
Mutation analysis of *eto1-16* and evolutionary analysis of ethylene overproduction 1 (ETO1). **(A)** Gene structure model of *ETO1*. The white box represents the untranslated region (UTR); the black box represents the protein-coding region; the black line represents the introns. Below the gene structure model, the mutant base site of *eto1-16* is shown, marked in red. **(B)** Protein domain structure of ETO1. Above the protein domain structure, the mutant amino acid site of *eto1-16* is shown, marked in red. **(C)** Multiple sequence alignment of the region around the mutation site in *eto1-16*. The red heart indicates the mutant position. **(D)** Protein sequence alignment of the region in **(C)** among ETO1, EOL1, and EOL2 in *Arabidopsis*. **(E)** Phylogenetic analysis of ETO1 among different species, including *Arabidopsis thaliana (At)*, *Zea mays (Zm)*, *Oryza sativa (Os)*, *Populus tomentosa (Pt)*, *Selaginella moellendorffii (Sm)*, and *Physcomitrella patens (Pp)*.

A map-based cloning approach was used to uncover the mutation site in *eto1-16*. The root phenotype of the F2 progeny (*eto1-16* × Landsberg erecta) segregated at an approximate *eto1-16* to WT ratio of 1:3, which suggests that *eto1-16* is a recessive mutation in a single locus (Supplementary Table S2). The mutation site was further mapped to chromosome 3 within a region containing *ETO1*. Afterward, the full-length genomic sequence of *ETO1*, including the 5'-UTR and 3'-UTR sequences, was amplified for sequencing. The sequencing results revealed a G to T transversion mutation in the first large exon at position 1,438 of the coding region (Figure 2A). Sequencing of other candidate genes did not reveal any additional mutations (data not shown).

Ethylene overproduction 1 is a highly conserved protein in plants. The *eto1-16* mutation caused a missense mutation from glycine to cysteine at position 480 in the linker sequence of TPR1 and TPR2 (Figure 2B; Supplementary Figure S2). The Gly480 is conserved in different plant species, including the lower plants *Selaginella moellendorffii* and *Physcomitrella patens* (Figure 2C). In *Arabidopsis*, ETO1 has two analogs, EOL1 and EOL2. Sequence analysis revealed that Gly480 is also conserved in both EOL1 and EOL2 (Figure 2D; Supplementary Figure S3). Notably, the linker region is long enough for the formation of a specific domain.

Previous studies have identified more than 20 *eto1* mutants, as mentioned above. Here, we systemically analyzed the *eto1* alleles, and found that six of them are amino acid substitution mutants, including *eto1-5*, *eto1-11*, *eto1-12*, *eto1-14*, *eto1-34*, and *hps3-2* (Supplementary Figure S4), while others have nonsense mutations that lead to premature termination codons. Except for *eto1-14*, which exhibits a substitution mutation in the BTB domain, the other five substitution mutations are located in the TPR motifs (Supplementary Figure S4). Mutations in *eto1-11* and *eto1-12* (in TPR1 and TPR5, respectively) are also caused by glycine substitutions of Gly450Arg and Gly779Glu, respectively. Both mutation sites are also conserved in the species examined (Supplementary Figures S2, S3). Furthermore, phylogenetic analysis revealed that ETO1 is highly conserved in different species (Figure 2E).

### Genetic and Biochemical Analysis of *eto1-16* Mutant

**Figure 3:**
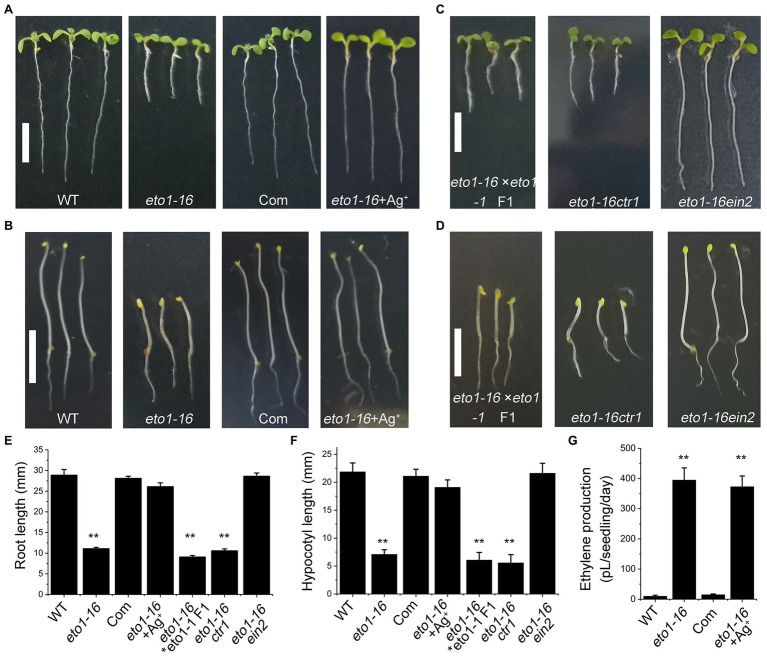
Genetic and biochemical analysis of *eto1-16*. **(A)** Root phenotypes of the WT, *eto1-16*, complementary (Com) and *eto1-16* (+Ag^+^) plants. Scale bar = 10 mm. **(B)** Hypocotyl phenotypes of the WT, *eto1-16*, Com, and *eto1-16* (+Ag^+^) plants. Scale bar = 10 mm. **(C)** Root phenotypes of F1 generation of cross line between *eto1-16* and *eto1-1*, and double mutants *eto1-16ctr1* and *eto1-16ein2*. Scale bar = 10 mm. **(D)** Hypocotyl phenotypes of F1 generation of cross line between *eto1-16* and *eto1-1*, and double mutants *eto1-16ctr1* and *eto1-16ein2*. Scale bar = 10 mm. **(E)** The root length of plants in **(A,B)**. **(F)** The hypocotyl length of plants in **(C,D)**. **(G)** Ethylene production in WT, *eto1-16*, Com, and *eto1-16* (+Ag^+^) plants. Statistical analysis was performed using a two-tailed Student’s *t*-test (^**^p < 0.01). For each experiment, at least three repeats were carried out in ethylene production test, and 50 plants were used in root and hypocotyl phenotypes tests.

To further confirm the mutation site in *eto1-16*, a complementary experiment was carried out by transforming the full-length WT *ETO1* into *eto1-16*. The root and hypocotyl phenotypes of the transgenic plants were rescued to those of the WT phenotype (Figures 3A,B,E,F). We further performed an allelic analysis by crossing *eto1-16* with *eto1-1*, a nonsense mutant. The F1 generation plants of the cross exhibited root and hypocotyl phenotypes similar to those of *eto1-1* (Figures 3C–F), which also indicated that *eto1-16* is a novel *eto1* allele.

Further analysis was carried out by crossing *eto1-16* with two ethylene signaling mutants, *ctr1* and *ein2*. CTR1 and EIN2 are negative and positive regulators in ethylene signaling, respectively. The root and hypocotyl of *eto1-16ctr1* showed mutant phenotypes similar to those of *ctr1*, while those of *eto1-16ein2* exhibited WT phenotypes (Figures 3C–F). Moreover, treatment with Ag^+^, an inhibitor for ethylene perception, can also rescue the *eto1-16* mutant phenotype to the WT phenotype (Figures 3A,B,E,F). These results further confirm the upstream position of *eto1-16* in ethylene responses. In addition, ethylene production in *eto1-16* is much higher than that of the WT (Figure 3G). The complementary *ETO1* decreases ethylene to a normal level (Figure 3G).

### Expression Analysis and Secondary Structure Prediction of *eto1-16* mRNA

**Figure 4:**
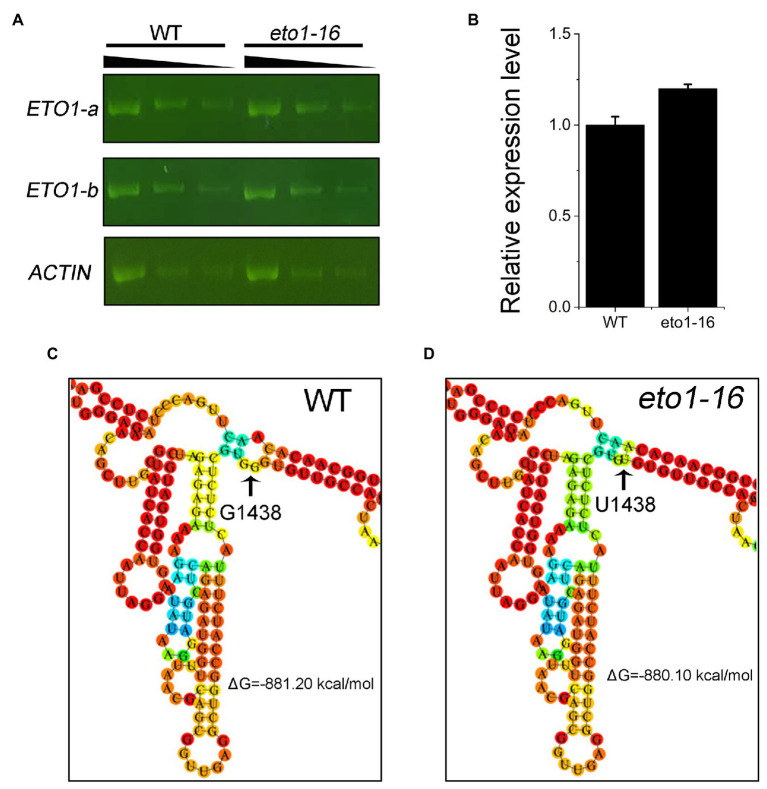
Expression level and RNA folding structure of WT and mutant *ETO1* transcripts. **(A)** Reverse transcription PCR (RT-PCR) results of *ETO1-a*, *ETO1-b*, and *Actin7* transcript fragments in WT and *eto1-16*. Black triangle (top) represents 3-fold serial dilutions of cDNA used for RT-PCR. **(B)** The relative expression levels of *ETO1* in WT and *eto1-16* quantified by quantitative real-time PCR (qRT-PCR). Partial predicted mRNA structure of *ETO1* transcripts of WT **(C)** and *eto1-16* **(D)**.

To further explain the effects of the *eto1-16* mutation, we analyzed the expression level of *ETO1* in 1-week-old WT and *eto1-16* plants by RT-PCR and qRT-PCR. The levels of *ETO1* transcripts in *eto1-16* were similar to those in WT (Figure 4A). The relative expression level of *ETO1* in *eto1-16* was similar to that in WT (Figure 4B). These results indicate that the mutation does not influence the *in vivo* mRNA abundance significantly.

The folding structures of *ETO1* mRNA in WT and *eto1-16* were further predicted using RNAfold 2.4.13. The overall mRNA secondary stem loop structure of *eto1-16* is similar to that of the WT (Figures 4C,D; Supplementary Figure S5). We also predicted the mRNA secondary stem loop structures of six missense mutation alleles, and a nonsense mutation, *eto1-1*. The *ETO1* mRNA structures of *eto1-5*, *eto1-11*, *eto1-12*, *eto1-34*, and *hps3-2* are similar to that of the WT, while ETO1 mRNA structures of *eto1-1* and *eto1-14* are the most different from that of the WT (Supplementary Figures S6–S9). These results indicate that the missense mutations in the TPR domain do not influence the mRNA structure and suggest that the mutant phenotype in *eto1-16* might be caused by the effects on ETO1 function.

### Biophysical Interaction Between ETO1 and ACSs Is Suppressed

**Figure 5:**
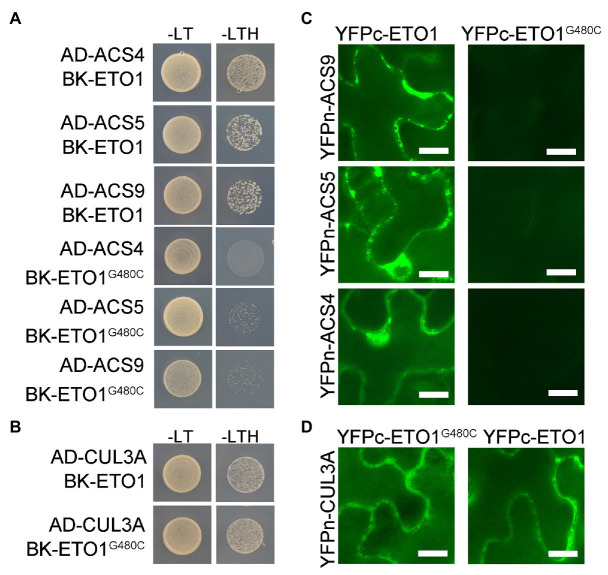
Interaction between ETO1 and type 2 1-aminocyclopropane-1-carboxylic acid (ACC) synthases (ACSs) is suppressed. **(A)** yeast two hybrid (Y2H) assay results between ETO1 and type 2 ACSs, including ACS4, ACS5, and ACS9. **(B)** Y2H results between ETO1 and CUL3A. **(C)** Bimolecular fluorescence complementation (BiFC) results between ETO1 and type 2 ACSs. **(D)** BiFC results of ETO1 and CUL3A. Bars = 10 μm.

To elucidate the effects of G480C mutation on ETO1 protein function, we investigated whether the mutation influences the physical interaction of ETO1 with ACSs and CUL3A proteins using Y2H assay. The WT ETO1 could interact with ACS4, ACS5, ACS9, and CUL3A, while the ETO1^G480C^ could not bind to ACS4, ACS5, and ACS9 (Figure 5A). However, ETO1^G480C^ could interact with CUL3A (Figure 5B), which was corroborated by the BiFC results (Figures 5C,D). The results indicated that Gly480 is crucial in the ETO1 and ACSs interaction although it is not required for the interaction with CUL3A. In addition, the suppression of the interaction between ETO1^G480C^ and ACSs might directly result in the abnormal root and hypocotyl development phenotypes in *eto1-16*.

Similarly, the Y2H results suggested that glycine substitution in *eto1-11* and *eto1-12* only affected the interaction between ACS5 and ETO1 but did not influence binding with CUL3A protein (Supplementary Figure S10B). Conversely, the *eto1-14* mutation disrupted the interaction of ETO1 with CUL3A, while interaction of ETO1 with ACSs was maintained (Supplementary Figure S10B). Binding of the BTB domain with CUL3A is not influenced by the *eto1-16* mutation within the TPR domain, and the mutation in the BTB domain does not affect the interaction of TPR with ACSs, which suggests that the two domains are partially independent.

### The Balance Between ETO1 and ACS5 Is Disturbed

**Figure 6:**
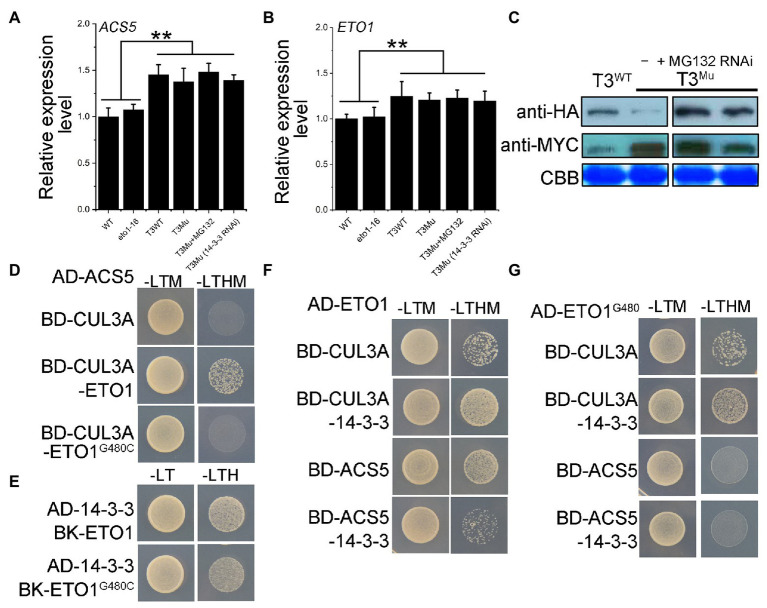
Expression levels of ACS5 in WT and *eto1-16* grown in a 16/8 h light/dark period, protein levels of HA-ETO1, HA-ETO1^G480C^, and MYC-ACS5 in genetically modified plants grown in a 16/8 h light/dark period and the regulatory relationship among ETO1, ACS5, 14-3-3, and CUL3A proteins. **(A)** *ACS5* expression levels in WT, *eto1-16*, and T3 plants quantified by qRT-PCR. Error bars represent SEs. Student’s *t*-test (^**^p < 0.01). **(B)** *ETO1* expression levels in WT, *eto1-16*, and T3 plants quantified by qRT-PCR. Error bars represent SEs. Student’s *t*-test (^**^p < 0.01). **(C)** Protein levels of HA-ETO1, HA-ETO1^G480C^, and ACS5 in plants T3^WT^, T3^Mu^, and T3^Mu^ following treatment of MG132 or 14-3-3 RNAi investigated by western blot. **(D)** Yeast three hybrid (Y3H) analysis by growth on auxotroph plate indicated the crucial role of ETO1 in linking the ACS5 and CUL3A. **(E)** Y2H analysis by growth on an auxotroph plate indicated the interaction of ETO1^G480C^ with 14-3-3. **(F)** Y3H assay among CUL3A, 14-3-3 and ETO1/ACS5 revealed the adverse roles of 14-3-3 in regulating ETO1 degradation and ACS5 stability. **(G)** Y3H assay results among CUL3A, 14-3-3, and ETO1^G480C^/ACS5.

The disruption of the protein-protein interaction between ETO1 and ACS5 releases the ETO1 and ACS5 from the complex, impairs the degradation of ACSs by the 26S proteasome, and results in cellular ACS accumulation (Wang et al., 2004; Christians et al., 2009). Due to a lack of antibodies for ETO1 and ACS5, to closely mimic the intracellular fate of the mutant ETO1 and its target ACS5 in *eto1-16*, we constructed a homozygous double mutant *eto1eto2* and introduced *MYC-ACS5* and *HA-ETO1^G480C^* transgenes using their native promoters (here named T3^Mu^) and using *eto1eto2* with transgenes *MYC-ACS5* and *HA-ETO1* (here named T3^WT^) as a control. The *ACS5* and *ETO1* expression levels in *eto1-16*, WT, T3^Mu^, and T3^WT^ were evaluated by qRT-PCR. The *ACS5* mRNA level showed no great difference between WT and *eto1-16* (Figures 6A,B; Supplementary Figure S11), while *ACS5* and *ETO1* mRNA levels in T3^Mu^ and T3^WT^ were about 1.4 and 1.2 times of those in *eto1-16* and WT, respectively, which is contributed by the double gene copy number in genetically modified plants. Furthermore, among T3^WT^, T3^Mu^, T3^Mu^ (MG132), and T3^Mu^ (14-3-3 RNAi) plants, no significant difference was observed in *ACS5* and *ETO1* mRNA abundance (Figures 6A,B; Supplementary Figure S11). Afterward, HA-ETO1, HA-ETO1^G480C^ and MYC-ACS5 levels in T3^Mu^ and T3^WT^ plants were detected using polyclonal antibodies for HA and MYC tags. MYC-ACS5 level increased approximately 10-fold in T3^Mu^ plants compared with that in T3^WT^ (Figure 6C). However, the HA-ETO1^G480C^ level in T3^Mu^ was much lower than that in T3^WT^. Treatment with MG132 of T3^Mu^ could rescue HA-ETO1^G480C^ to a high level (Figure 6C; Supplementary Figure S11), which indicates that the HA-ETO1^G480C^ is prone to be degraded *via* the 26S proteasome pathway. Similarly, in 14-3-3 RNAi T3^Mu^ plants, which inhibits the destabilization effects of 14-3-3 on ETO1, HA-ETO1^G480C^ level was also rescued to a level comparable to that of HA-ETO1 in T3^WT^ plants (Figure 6C). In contrast, treatment with MG132 showed no obvious influence on MYC-ACS5 level, while in 14-3-3 RNAi T3^Mu^ plants, MYC-ACS5 level was decreased by 50% (Figure 6C; Supplementary Figure S11). It is suggested that 14-3-3 has a partial participation in *ETO1^G480C^* degradation and MYC-ACS5 stabilization as previously reported.

To reveal the underlining regulation mechanism, we characterized the potential regulatory relationships among the proteins ETO1, ACS5, 14-3-3, and CUL3A using Y2H and Y3H assays. The results in Figure 6D demonstrate that ETO1 is a “linker” protein for CUL3A capturing ACS5. However, this link is disrupted by G480C mutation (Figure 6D), which explains ACS5 accumulation and further ACS5 stabilization by 14-3-3 in T3^Mu^ plants (Figure 6C). Y2H assay results showed that ETO1^G480C^ interacts with 14-3-3 (Figure 6E); moreover, 14-3-3 could enhance the binding ability of ETO1 with CUL3A to some extent (Figure 6F), which is still sound and might promote HA-ETO1^G480C^ degradation in T3^Mu^ plants (Figure 6C). In contrast, the binding of ETO1 to ACS5 is partially inhibited by 14-3-3 (Figure 6F), suggesting that ETO1 and 14-3-3 could bind competitively to ACS5. And the G480C mutation disrupted this competition (Figure 6G). These results not only highlight the potentially important role of the linker sequence between TPR1 and TPR2 for ETO1 in targeting ACSs for degradation but also indicate that the complex formation of ETO1 and ACSs, together with other regulators, including 14-3-3 and CUL3A facilitate the maintenance of a balance in the ETO1 and ACS protein pools.

**Figure 7:**
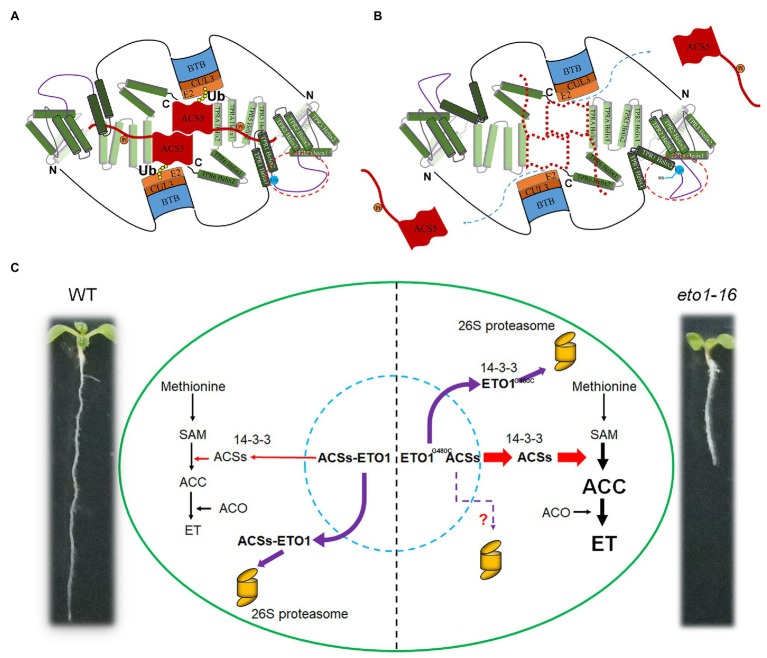
Schematic binding model of ETO1 with ACS5 and pool of ETO1 and ACS5 in WT and *eto1-16*. **(A)** Tetratricopeptide repeat (TPR) motifs of ETO1 bind to the extended C-terminus of ACS5 in WT. The dimerization of ACS5 and phosphorylation (orange circles labeled with “Pi”) enhance the binding of ACS5 to ETO1. Ubiquitination (Ub) is indicated as yellow filled circles. Except for the BTB domain and TPR motifs, other regions are represented as lines. The linker sequence between TPR1 and TPR2 is encircled by red dotted lines, and the mutation site is labeled by blue filled circles. “N” and “C” represent the N terminus and C terminus of ETO1, respectively. **(B)** The potential conformation change in linker sequence caused by *eto1-16* mutation disrupted the protein interaction between ETO1 and ACS5 and released ACS5 from the complex. **(C)** Regulation of ACS5 activity by ETO1 in WT and *eto1-16*. ETO1 interacts with ACS5, inhibits its enzyme activity, and targets it for proteasome-dependent degradation *via* interaction with the scaffold subunit CUL3A. In *eto1-16*, disruption of the interaction between ETO1 and ACS5 releases them from the complex. The ETO1 is degraded by the 26S proteasome, whereas the functional ACS5 catalyzes the S-adenosylmethionine (SAM) to ACC conversion.

The TPR motif is a typical module in protein-protein interactions of 34 amino acids with the consensus sequences four (W/L/F), seven (L/I/M), eight (G/A/S), 11 (Y/L/F), 20 (A/S/E), 24 (F/Y/L), 27 (A/S/L), and 32 (P/K/E). There are also seven TPR motifs in ETO1 (Figure 2B; Supplementary Figures S2, S4), which bind to a C-terminal extend motif of ACSs (Supplementary Figure S12). Contrastingly, the linker sequence between the adjacent TPRs is extensive, especially between TPR1 and TPR2 (Figures 2, 7A,B; Supplementary Figure S2). The abnormal phenotypes of *eto1-5*, *eto1-11*, *eto1-12*, *eto1-34*, and *hps3-2* mutants indicated that TPR1, TPR5, and TPRA play important roles in binding with ACSs (Figure 7A; Supplementary Figure S2). *eto1-11* and *eto1-12* mutation sites were located at position eight (glycine) of helix 1 of TPR1 and TPR5, respectively (Supplementary Figures S2, S4). In contrast, the mutation site of *eto1-16* located at the linker sequence of TPR1 and TPR2 might alter the structure of the linker region and/or the orientation of TPR motifs (Figure 7B). To investigate the potential effect of *eto1-16* on the ETO1 conformation, we used this linker sequence to search the similar model structure in the PDB database. The result showed that the sequence was aligned to the helix part of the TPR containing proteins with similarities ranging from 14 to 24% (Supplementary Figure S13), which indicates the potential helix formation of this linker region. Further investigation of the helix formation ability of the first 15 AA showed that mutation of Gly480 to Cys could greatly disrupt its amphipathicity (Supplementary Figure S13). It is suggested that the uncharacterized regions of ETO1 are also key elements in the regulation of ACS stability. For example, it could provide a flexible framework for the appropriate ETO1 conformation as previously reported (Stanley et al., 2007).

## Discussion

In this study, we isolated a novel *eto1* allele with typical triple response phenotypes of shortened and swollen hypocotyls, exaggerated apical hooks, and short roots with excessive root hairs that is observed in ethylene biosynthesis and signaling defective mutants, such as *eto1*, *eto2*, *eto3*, and *ctr1* (Guzman and Ecker, 1990; Kieber et al., 1993; Chae et al., 2003). Based on the phenotype analysis and mapping results, *ETO1* was selected as the mutation candidate for sequencing. The sequencing results confirmed the ETO1 mutation of G1438T, which results in an amino acid Gly-to-Cys missense mutation in the linker region between TPR1 and TPR2. However, there are no similar reports to probe the potential regulatory role of the linker region between the TPR motifs. Our genetic and biochemical experiments further verified that the site mutation in *eto1-16* leads to the emergence of the triple response phenotypes and high ethylene production. High ethylene concentration can induce asymmetric auxin redistribution in *eto1* mutants, which directly influences root epidermal cell elongation and root hair elongation (Ruzicka et al., 2007; Sun et al., 2010). Similarly, the PIN2:GFP fusion protein was increased in *eto1-16* compared to that in WT (Supplementary Figure S14).

Similar to *eto1-16*, previously identified *eto1-11* and *eto1-12* mutations also have a glycine substitution (Ortega-Martinez et al., 2007). These three mutation sites did not influence mRNA abundance of *ETO1* and *ACS5* but disrupted the interaction of ETO1 and ACS proteins (Figures 5A,C). Compared with glycine, cysteine has a side chain with an active sulfhydryl group. Similarly, in *eto1-11* and *eto1-12* mutants, glutamic acid and arginine, respectively, have large side chains with amide groups. Glycine is the smallest building block in protein synthesis and the only one without chirality. According to the results of the present study and a previous study (Ortega-Martinez et al., 2007), the small-sized glycine in the linker region between or within the TPR motifs is essential for ETO1 function and might play roles in fine-tuning the flexible TPR structure for rapidly changeable protein-protein interactions between ETO1 and various regulators. The conservation of the corresponding glycine in different species and EOL1 and EOL2 highlight the important role of the site in EOL1 and EOL2 (Supplementary Figures S2, S3).

Ethylene overproduction 1 has several characterized domains (Figure 2). Except for the above mentioned, the N-terminal BTB domain and the C-terminal TPR domain, there are proline-rich and glycine-rich domains with unknown functions on the extreme N terminus and a CC-domain between TPR5 and TPR6. The alignment of the full-length protein in several species revealed that ETO1 is a highly conserved protein, especially the C-terminal TPR domain (Supplementary Figure S2). In contrast, the G-rich and P-rich domains are not conserved in the species examined. In *Populus*, there is no P-rich domain, while in monocotyledons, the order of the two domains is reversed. Both the G-rich and P-rich domains are lost in lower plants *S. moellendorffii* and *P. patens* (Supplementary Figure S2). The two paralogs, EOL1 and EOL2, also do not have these two N-terminal domains (Supplementary Figure S3). These results suggest that the two domains are not essential for ETO1 function. The *eto1-16* mutation site is located in an uncharacterized linker region between TPR1 and TPR2, which indicates the essential role of the linker sequence for ETO1 function. The TPR motif is found in multiple copies in many functionally different proteins and is often arranged in tandem arrays (D’andrea and Regan, 2003; Perez-Riba and Itzhaki, 2019). For example, seven TPR motifs of PEX5 form an antiparallel helix-turn-helix unit and have a role in the recognition and binding of the C-terminal “SKL” tripeptides, or variants thereof, of ligand proteins (Sampathkumar et al., 2008). Similarly, the mutation of glycine 8 in PEX5 can disrupt its ligand recognition ability (Patel et al., 2019). This indicates a potentially similar TPR structure formation between ETO1 and PEX5.

In *Arabidopsis*, EOL1 and EOL2 amplify the effects of ETO1 inactivation and further increase ethylene production and ACS5 accumulation in *eto1* plants (Christians et al., 2009). However, single and double mutants affecting EOL1 and EOL2 do not exhibit an ethylene-related triple phenotype (Christians et al., 2009). Therefore, ETO1 is considered the primary regulator of ACSs stability, while EOL1 and EOL2 might play a role as auxiliary regulators or respond to stresses distinct from those responded by ETO1. In addition, the conservation intensity of ETO1 protein in several diverse related species is higher than those of EOL1 and EOL2 (Supplementary Figures S2, S3), which also suggests that the accumulation of mutation sites of EOL1 and EOL2 (in comparison with ETO1) might be the cause of their loss of function in the triple responses.

Different C-terminal domains among three classes of ACS proteins impart distinct regulatory controls on the stability of the respective ACS proteins (Spanu et al., 1994; Yamagami et al., 2003; Chae and Kieber, 2005; Polko and Kieber, 2019). ETO1, EOL1, and EOL2 specifically interact with the C terminus of the type 2 ACS subfamily, but not with the other two types of ACSs (Christians et al., 2009). Constitutive expression of ETO1 results in post-transcriptional suppression of a type 2 ACC synthase (Wang et al., 2004). Moreover, phosphorylation of ACS5 by CK1.8 can enhance its binding activity with ETO1 and target it to degradation (Tan and Xue, 2014). Conversely, 14-3-3 can interact directly with ETO1, EOL1, and EOL2 to destabilize them, whereas the stability of both type 2 and type 3 ACS proteins is enhanced by interaction with 14-3-3 (Yoon and Kieber, 2013). However, a detailed interaction model of ETO1 with both ACSs, CUL3A, and some auxiliary regulators has not been established.

The TPR domain of ETO1 is extensive, so that ETO1 with different TPR conformations might interact with different regulators, which could be altered rapidly, according to the environmental cues. Here, we presume that ETO1 occurs as a multi-state pool, with ETO1 having several different forms, such as ETO1 monomers, ETO1-ACSs, ETO1-CUL3A complexes, and the ETO1-CUL3A-ACSs super complex (Figure 7). We also established a regulation model. In WT, ETO1 and ACS5 protein pools maintain a balance *via* various processes, such as phosphorylation, dephosphorylation, and interaction with 14-3-3 (Figure 7C). And, the ETO1-ACS5 complex is the dominant form, which targets ACS5 degradation and consequently limits the concentrations of free forms of ACS5 to control the rate of the conversion of SAM into ACC. In *eto1* mutants, which exhibited disrupted ETO1 and ACS5 interactions, such as *eto1-16*, more active ACS5 are released to catalyze ACC production, and ultimately, high ethylene amounts are produced and downstream gene events are induced, resulting in the defective root phenotype (Figure 7C). In addition, the cytosolic 14-3-3 proteins further stabilize the released ACS5 but destabilize the mutant ETO1 by promoting its degradation. Although the genetically manipulated plants were not equivalent to *eto1-16*, the results could reflect the protein-protein interactions in *eto1-16* to some extent. According to our results, the *eto1-16* mutant has low level of ETO1^G480C^ proteins due to lack of binding with ACSs, which promotes more ETO1^G480C^ to interact with 14-3-3 and CUL3A, as well as ETO1 destabilization and degradation by the 26S proteasome. However, it is inexplicable that the role of 14-3-3 and CUL3A in HA-ETO^G480C^ degradation is not reflected by the protein-protein interaction analysis, which indicated that G480C mutation have some other potential effects on ETO1, such as conformation change.

The synergistic effects of ethylene and other phytohormones, such as auxin, ABA, JA, cytokinin, and gibberellins are well defined in the regulation of fruit ripening, root growth and gravitropism, root hair growth and differentiation, hypocotyl elongation, and apical hook formation (Van de Poel et al., 2015; Qin et al., 2019; An et al., 2020; Xu et al., 2020), suggesting that they also interact at the molecular level. ETO1 could also be a key modulator of such synergistic processes. Besides 14-3-3 and CK1.8, other unidentified regulation proteins, which also interact with ETO1 (especially for the uncharacterized linker region in TPR domain), need to be further isolated and characterized to elucidate the comprehensive ETO1 function map and provide a better understanding of the role of ethylene in response to *in vivo* or *in vitro* environmental stimulus.

## Data Availability Statement

The datasets presented in this study can be found in online repositories. The names of the repository/repositories and accession number(s) can be found in the article/ Supplementary Material.

## Author Contributions

CA designed the research. CA and YG performed the experiments, analyzed the data, and wrote the manuscript. Both the authors contributed to the article and approved the submitted version.

### Conflict of Interest

The authors declare that the research was conducted in the absence of any commercial or financial relationships that could be construed as a potential conflict of interest.

## Footnotes

^1^www.arabidopsis.org/Blast/index.jsp

^2^www.ncbi.nlm.nih.gov/guide/homology/

^3^http://rna.tbi.univie.ac.at/cgi-bin/RNAWebSuite/RNAfold.cgi

^4^https://heliquest.ipmc.cnrs.fr/

^5^https://www.expasy.org/resources/swiss-model

## References

[ref1] Abel S. Nguyen M. D. Chow W. Theologis A. (1995). *ACS4*, a primary indoleacetic acid-responsive gene encoding 1-aminocyclopropane-1-carboxylate synthase in *Arabidopsis thaliana*. Structural characterization, expression in *Escherichia coli*, and expression characteristics in response to auxin [corrected]. J. Biol. Chem. 270, 19093–19099. 10.1074/jbc.270.32.19093, PMID: 7642574

[ref2] Ahammed G. J. Gantait S. Mitra M. Yang Y. Li X. (2020). Role of ethylene crosstalk in seed germination and early seedling development: a review. Plant Physiol. Biochem. 151, 124–131. 10.1016/j.plaphy.2020.03.016, PMID: 32220785

[ref3] Alonso J. M. Hirayama T. Roman G. Nourizadeh S. Ecker J. R. (1999). EIN2, a bifunctional transducer of ethylene and stress responses in *Arabidopsis*. Science 284, 2148–2152. 10.1126/science.284.5423.2148, PMID: 10381874

[ref4] An J. Althiab Almasaud R. Bouzayen M. Zouine M. Chervin C. (2020). Auxin and ethylene regulation of fruit set. Plant Sci. 292:110381. 10.1016/j.plantsci.2019.110381, PMID: 32005386

[ref5] An C. Gao Y. Li J. Liu X. Gao F. Gao H. (2017). Alternative splicing affects the targeting sequence of peroxisome proteins in *Arabidopsis*. Plant Cell Rep. 36, 1027–1036. 10.1007/s00299-017-2131-2, PMID: 28352967

[ref6] Bakshi A. Shemansky J. M. Chang C. R. Binder B. M. (2015). History of research on the plant hormone ethylene. J. Plant Growth Regul. 34, 809–827. 10.1007/s00344-015-9522-9

[ref7] Binder B. M. (2020). Ethylene signaling in plants. J. Biol. Chem. 295, 7710–7725. 10.1074/jbc.REV120.010854, PMID: 32332098 PMC7261785

[ref8] Chae H. S. Faure F. Kieber J. J. (2003). The *eto1*, *eto2*, and *eto3* mutations and cytokinin treatment increase ethylene biosynthesis in *Arabidopsis* by increasing the stability of ACS protein. Plant Cell 15, 545–559. 10.1105/tpc.006882, PMID: 12566591 PMC141220

[ref9] Chae H. S. Kieber J. J. (2005). Eto Brute? Role of ACS turnover in regulating ethylene biosynthesis. Trends Plant Sci. 10, 291–296. 10.1016/j.tplants.2005.04.006, PMID: 15949763

[ref10] Chen J. Wang X. Zhang W. Zhang S. Zhao F. J. (2020). Protein phosphatase 2A alleviates cadmium toxicity by modulating ethylene production in *Arabidopsis thaliana*. Plant Cell Environ. 43, 1008–1022. 10.1111/pce.13716, PMID: 31916592

[ref11] Christians M. J. Gingerich D. J. Hansen M. Binder B. M. Kieber J. J. Vierstra R. D. (2009). The BTB ubiquitin ligases ETO1, EOL1 and EOL2 act collectively to regulate ethylene biosynthesis in *Arabidopsis* by controlling type-2 ACC synthase levels. Plant J. 57, 332–345. 10.1111/j.1365-313X.2008.03693.x, PMID: 18808454 PMC2807402

[ref12] D’andrea L. D. Regan L. (2003). TPR proteins: the versatile helix. Trends Biochem. Sci. 28, 655–662. 10.1016/j.tibs.2003.10.007, PMID: 14659697

[ref13] Dolgikh V. A. Pukhovaya E. M. Zemlyanskaya E. V. (2019). Shaping ethylene response: the role of EIN3/EIL1 transcription factors. Front. Plant Sci. 10:1030. 10.3389/fpls.2019.01030, PMID: 31507622 PMC6718143

[ref14] Dubois M. Van den Broeck L. Inze D. (2018). The pivotal role of ethylene in plant growth. Trends Plant Sci. 23, 311–323. 10.1016/j.tplants.2018.01.003, PMID: 29428350 PMC5890734

[ref15] Ecker J. R. (1995). The ethylene signal transduction pathway in plants. Science 268, 667–675. 10.1126/science.7732375, PMID: 7732375

[ref16] Grefen C. Stadele K. Ruzicka K. Obrdlik P. Harter K. Horak J. (2008). Subcellular localization and in vivo interactions of the *Arabidopsis thaliana* ethylene receptor family members. Mol. Plant 1, 308–320. 10.1093/mp/ssm015, PMID: 19825542

[ref17] Guzman P. Ecker J. R. (1990). Exploiting the triple response of *Arabidopsis* to identify ethylene-related mutants. Plant Cell 2, 513–523. 10.1105/tpc.2.6.513, PMID: 2152173 PMC159907

[ref18] Habben J. E. Bao X. Bate N. J. Debruin J. L. Dolan D. Hasegawa D.. (2014). Transgenic alteration of ethylene biosynthesis increases grain yield in maize under field drought-stress conditions. Plant Biotechnol. J. 12, 685–693. 10.1111/pbi.12172, PMID: 24618117

[ref19] Hartman S. Sasidharan R. Voesenek L. (2021). The role of ethylene in metabolic acclimations to low oxygen. New Phytol. 229, 64–70. 10.1111/nph.16378, PMID: 31856295 PMC7754284

[ref20] Hou X. Li L. Peng Z. Wei B. Tang S. Ding M.. (2010). A platform of high-density INDEL/CAPS markers for map-based cloning in *Arabidopsis*. Plant J. 63, 880–888. 10.1111/j.1365-313X.2010.04277.x, PMID: 20561258

[ref21] Huang G. Zhang D. (2020). The plasticity of root systems in response to external phosphate. Int. J. Mol. Sci. 21:5955. 10.3390/ijms21175955, PMID: 32824996 PMC7503333

[ref22] Ju C. Chang C. (2015). Mechanistic insights in ethylene perception and signal transduction. Plant Physiol. 169, 85–95. 10.1104/pp.15.00845, PMID: 26246449 PMC4577421

[ref23] Ju C. Yoon G. M. Shemansky J. M. Lin D. Y. Ying Z. I. Chang J.. (2012). CTR1 phosphorylates the central regulator EIN2 to control ethylene hormone signaling from the ER membrane to the nucleus in *Arabidopsis*. Proc. Natl. Acad. Sci. U. S. A. 109, 19486–19491. 10.1073/pnas.1214848109, PMID: 23132950 PMC3511113

[ref24] Kamiyoshihara Y. Iwata M. Fukaya T. Tatsuki M. Mori H. (2010). Turnover of LeACS2, a wound-inducible 1-aminocyclopropane-1-carboxylic acid synthase in tomato, is regulated by phosphorylation/dephosphorylation. Plant J. 64, 140–150. 10.1111/j.1365-313X.2010.04316.x, PMID: 20659278

[ref25] Kieber J. J. Rothenberg M. Roman G. Feldmann K. A. Ecker J. R. (1993). CTR1, a negative regulator of the ethylene response pathway in *Arabidopsis*, encodes a member of the raf family of protein kinases. Cell 72, 427–441. 10.1016/0092-8674(93)90119-B, PMID: 8431946

[ref26] Kumar S. Stecher G. Li M. Knyaz C. Tamura K. (2018). MEGA X: molecular evolutionary genetics analysis across computing platforms. Mol. Biol. Evol. 35, 1547–1549. 10.1093/molbev/msy096, PMID: 29722887 PMC5967553

[ref27] Lei M. G. Zhu C. M. Liu Y. D. Karthikeyan A. S. Bressan R. A. Raghothama K. G.. (2011). Ethylene signalling is involved in regulation of phosphate starvation-induced gene expression and production of acid phosphatases and anthocyanin in *Arabidopsis*. New Phytol. 189, 1084–1095. 10.1111/j.1469-8137.2010.03555.x, PMID: 21118263

[ref28] Li S. Chen K. Grierson D. (2019). A critical evaluation of the role of ethylene and MADS transcription factors in the network controlling fleshy fruit ripening. New Phytol. 221, 1724–1741. 10.1111/nph.15545, PMID: 30328615

[ref29] Li W. Ma M. Feng Y. Li H. Wang Y. Ma Y.. (2015). EIN2-directed translational regulation of ethylene signaling in *Arabidopsis*. Cell 163, 670–683. 10.1016/j.cell.2015.09.037, PMID: 26496607

[ref30] Liang X. Oono Y. Shen N. F. Kohler C. Li K. Scolnik P. A.. (1995). Characterization of two members (ACS1 and ACS3) of the 1-aminocyclopropane-1-carboxylate synthase gene family of *Arabidopsis thaliana*. Gene 167, 17–24. 10.1016/0378-1119(95)00694-X, PMID: 8566772

[ref31] Lin Z. Zhong S. Grierson D. (2009). Recent advances in ethylene research. J. Exp. Bot. 60, 3311–3336. 10.1093/jxb/erp204, PMID: 19567479

[ref32] Liu M. Pirrello J. Chervin C. Roustan J. P. Bouzayen M. (2015). Ethylene control of fruit ripening: revisiting the complex network of transcriptional regulation. Plant Physiol. 169, 2380–2390. 10.1104/pp.15.01361, PMID: 26511917 PMC4677914

[ref33] Liu Y. Zhang S. (2004). Phosphorylation of 1-aminocyclopropane-1-carboxylic acid synthase by MPK6, a stress-responsive mitogen-activated protein kinase, induces ethylene biosynthesis in *Arabidopsis*. Plant Cell 16, 3386–3399. 10.1105/tpc.104.026609, PMID: 15539472 PMC535880

[ref34] Lyzenga W. J. Booth J. K. Stone S. L. (2012). The *Arabidopsis* RING-type E3 ligase XBAT32 mediates the proteasomal degradation of the ethylene biosynthetic enzyme, 1-aminocyclopropane-1-carboxylate synthase 7. Plant J. 71, 23–34. 10.1111/j.1365-313X.2012.04965.x, PMID: 22339729

[ref35] Mayerhofer H. Panneerselvam S. Mueller-Dieckmann J. (2012). Protein kinase domain of CTR1 from *Arabidopsis thaliana* promotes ethylene receptor cross talk. J. Mol. Biol. 415, 768–779. 10.1016/j.jmb.2011.11.046, PMID: 22155294

[ref36] Merchante C. Alonso J. M. Stepanova A. N. (2013). Ethylene signaling: simple ligand, complex regulation. Curr. Opin. Plant Biol. 16, 554–560. 10.1016/j.pbi.2013.08.001, PMID: 24012247

[ref37] Merchante C. Brumos J. Yun J. Hu Q. Spencer K. R. Enriquez P.. (2015). Gene-specific translation regulation mediated by the hormone-signaling molecule EIN2. Cell 163, 684–697. 10.1016/j.cell.2015.09.036, PMID: 26496608

[ref38] Ortega-Martinez O. Pernas M. Carol R. J. Dolan L. (2007). Ethylene modulates stem cell division in the *Arabidopsis thaliana* root. Science 317, 507–510. 10.1126/science.1143409, PMID: 17656722

[ref39] Pan C. Zhang H. Ma Q. Fan F. Fu R. Ahammed G. J.. (2019). Role of ethylene biosynthesis and signaling in elevated CO_2_-induced heat stress response in tomato. Planta 250, 563–572. 10.1007/s00425-019-03192-5, PMID: 31123806

[ref40] Patel K. J. Kao Y. T. Llinas R. J. Bartel B. (2019). A PEX5 missense allele preferentially disrupts PTS1 cargo import into *Arabidopsis* peroxisomes. Plant Direct. 3:e00128. 10.1002/pld3.128, PMID: 31236542 PMC6508846

[ref41] Perata P. (2020). Ethylene signaling controls fast oxygen sensing in plants. Trends Plant Sci. 25, 3–6. 10.1016/j.tplants.2019.10.010, PMID: 31734094

[ref42] Perez-Riba A. Itzhaki L. S. (2019). The tetratricopeptide-repeat motif is a versatile platform that enables diverse modes of molecular recognition. Curr. Opin. Struct. Biol. 54, 43–49. 10.1016/j.sbi.2018.12.004, PMID: 30708253

[ref43] Polko J. K. Kieber J. J. (2019). 1-Aminocyclopropane 1-carboxylic acid and its emerging role as an ethylene-independent growth regulator. Front. Plant Sci. 10:1602. 10.3389/fpls.2019.01602, PMID: 31921251 PMC6915048

[ref44] Potuschak T. Lechner E. Parmentier Y. Yanagisawa S. Grava S. Koncz C.. (2003). EIN3-dependent regulation of plant ethylene hormone signaling by two *Arabidopsis* F box proteins: EBF1 and EBF2. Cell 115, 679–689. 10.1016/S0092-8674(03)00968-1, PMID: 14675533

[ref45] Qin H. He L. Huang R. (2019). The coordination of ethylene and other hormones in primary root development. Front. Plant Sci. 10:874. 10.3389/fpls.2019.00874, PMID: 31354757 PMC6635467

[ref46] Rodrigues M. A. Bianchetti R. E. Freschi L. (2014). Shedding light on ethylene metabolism in higher plants. Front. Plant Sci. 5:665. 10.3389/fpls.2014.00665, PMID: 25520728 PMC4249713

[ref47] Ruzicka K. Ljung K. Vanneste S. Podhorska R. Beeckman T. Friml J.. (2007). Ethylene regulates root growth through effects on auxin biosynthesis and transport-dependent auxin distribution. Plant Cell 19, 2197–2212. 10.1105/tpc.107.052126, PMID: 17630274 PMC1955700

[ref48] Sampathkumar P. Roach C. Michels P. A. Hol W. G. (2008). Structural insights into the recognition of peroxisomal targeting signal 1 by Trypanosoma brucei peroxin 5. J. Mol. Biol. 381, 867–880. 10.1016/j.jmb.2008.05.089, PMID: 18598704

[ref49] Seo D. H. Yoon G. M. (2019). Light-induced stabilization of ACS contributes to hypocotyl elongation during the dark-to-light transition in *Arabidopsis* seedlings. Plant J. 98, 898–911. 10.1111/tpj.14289, PMID: 30776167

[ref50] Sievers F. Wilm A. Dineen D. Gibson T. J. Karplus K. Li W. Z.. (2011). Fast, scalable generation of high-quality protein multiple sequence alignments using Clustal Omega. Mol. Syst. Biol. 7:539. 10.1038/msb.2011.75, PMID: 21988835 PMC3261699

[ref51] Skottke K. R. Yoon G. M. Kieber J. J. Delong A. (2011). Protein phosphatase 2A controls ethylene biosynthesis by differentially regulating the turnover of ACC synthase isoforms. PLoS Genet. 7:e1001370. 10.1371/journal.pgen.1001370, PMID: 21533019 PMC3080859

[ref52] Spanu P. Grosskopf D. G. Felix G. Boller T. (1994). The apparent turnover of 1-aminocyclopropane-1-carboxylate synthase in tomato cells is regulated by protein phosphorylation and dephosphorylation. Plant Physiol. 106, 529–535. 10.1104/pp.106.2.529, PMID: 12232347 PMC159558

[ref53] Stanley W. A. Pursiainen N. V. Garman E. F. Juffer A. H. Wilmanns M. Kursula P. (2007). A previously unobserved conformation for the human Pex5p receptor suggests roles for intrinsic flexibility and rigid domain motions in ligand binding. BMC Struct. Biol. 7:24. 10.1186/1472-6807-7-24, PMID: 17428317 PMC1854907

[ref54] Steffens B. (2014). The role of ethylene and ROS in salinity, heavy metal, and flooding responses in rice. Front. Plant Sci. 5:685. 10.3389/fpls.2014.00685, PMID: 25538719 PMC4255495

[ref55] Sun P. Tian Q. Y. Chen J. Zhang W. H. (2010). Aluminium-induced inhibition of root elongation in *Arabidopsis* is mediated by ethylene and auxin. J. Exp. Bot. 61, 347–356. 10.1093/jxb/erp306, PMID: 19858117 PMC2803203

[ref56] Tan S. T. Xue H. W. (2014). Casein kinase 1 regulates ethylene synthesis by phosphorylating and promoting the turnover of ACS5. Cell Rep. 9, 1692–1702. 10.1016/j.celrep.2014.10.047, PMID: 25464840

[ref57] Tsuchisaka A. Yu G. Jin H. Alonso J. M. Ecker J. R. Zhang X.. (2009). A combinatorial interplay among the 1-aminocyclopropane-1-carboxylate isoforms regulates ethylene biosynthesis in *Arabidopsis thaliana*. Genetics 183, 979–1003. 10.1534/genetics.109.107102, PMID: 19752216 PMC2778992

[ref58] Van de Poel B. Smet D. Van Der Straeten D. (2015). Ethylene and hormonal cross talk in vegetative growth and development. Plant Physiol. 169, 61–72. 10.1104/pp.15.00724, PMID: 26232489 PMC4577414

[ref59] Vogel J. P. Woeste K. E. Theologis A. Kieber J. J. (1998). Recessive and dominant mutations in the ethylene biosynthetic gene ACS5 of *Arabidopsis* confer cytokinin insensitivity and ethylene overproduction, respectively. Proc. Natl. Acad. Sci. U. S. A. 95, 4766–4771. 9539813 10.1073/pnas.95.8.4766 PMC22565

[ref60] Wang F. H. Ahammed G. J. Li G. Y. Bai P. T. Jiang Y. Wang S. X.. (2019). Ethylene is involved in red light-induced anthocyanin biosynthesis in cabbage (*Brassica oleracea*). Int. J. Agric. Biol. 21, 955–963. 10.17957/IJAB/15.0980

[ref61] Wang K. L. Yoshida H. Lurin C. Ecker J. R. (2004). Regulation of ethylene gas biosynthesis by the *Arabidopsis* ETO1 protein. Nature 428, 945–950. 10.1038/nature02516, PMID: 15118728

[ref62] Woeste K. E. Ye C. Kieber J. J. (1999). Two *Arabidopsis* mutants that overproduce ethylene are affected in the posttranscriptional regulation of 1-aminocyclopropane-1-carboxylic acid synthase. Plant Physiol. 119, 521–530. 10.1104/pp.119.2.521, PMID: 9952448 PMC32129

[ref63] Xu P. Zhao P. X. Cai X. T. Mao J. L. Miao Z. Q. Xiang C. B. (2020). Integration of jasmonic acid and ethylene into auxin signaling in root development. Front. Plant Sci. 11:271. 10.3389/fpls.2020.00271, PMID: 32211015 PMC7076161

[ref64] Yamagami T. Tsuchisaka A. Yamada K. Haddon W. F. Harden L. A. Theologis A. (2003). Biochemical diversity among the 1-amino-cyclopropane-1-carboxylate synthase isozymes encoded by the *Arabidopsis* gene family. J. Biol. Chem. 278, 49102–49112. 10.1074/jbc.M308297200, PMID: 12968022

[ref65] Yang S. F. Hoffman N. E. (1984). Ethylene biosynthesis and its regulation in higher plants. Annu. Rev. Plant Physiol. 35, 155–189. 10.1146/annurev.pp.35.060184.001103

[ref66] Yasumura Y. Pierik R. Kelly S. Sakuta M. Voesenek L. A. Harberd N. P. (2015). An ancestral role for CONSTITUTIVE TRIPLE RESPONSE1 proteins in both ethylene and abscisic acid signaling. Plant Physiol. 169, 283–298. 10.1104/pp.15.00233, PMID: 26243614 PMC4577374

[ref67] Yoon G. M. Kieber J. J. (2013). 14-3-3 regulates 1-aminocyclopropane-1-carboxylate synthase protein turnover in *Arabidopsis*. Plant Cell 25, 1016–1028. 10.1105/tpc.113.110106, PMID: 23512855 PMC3634674

[ref68] Yoshida H. Nagata M. Saito K. Wang K. L. Ecker J. R. (2005). *Arabidopsis* ETO1 specifically interacts with and negatively regulates type 2 1-aminocyclopropane-1-carboxylate synthases. BMC Plant Biol. 5:14. 10.1186/1471-2229-5-14, PMID: 16091151 PMC1199607

[ref69] Yoshida H. Wang K. L. Chang C. M. Mori K. Uchida E. Ecker J. R. (2006). The ACC synthase TOE sequence is required for interaction with ETO1 family proteins and destabilization of target proteins. Plant Mol. Biol. 62, 427–437. 10.1007/s11103-006-9029-7, PMID: 16897471

[ref70] Zhang F. Wang L. Qi B. Zhao B. Ko E. E. Riggan N. D.. (2017). EIN2 mediates direct regulation of histone acetylation in the ethylene response. Proc. Natl. Acad. Sci. U. S. A. 114, 10274–10279. 10.1073/pnas.1707937114, PMID: 28874528 PMC5617289

[ref71] Zhao H. Yin C. C. Ma B. Chen S. Y. Zhang J. S. (2021). Ethylene signaling in rice and *Arabidopsis*: new regulators and mechanisms. J. Integr. Plant Biol. 63, 102–125. 10.1111/jipb.13028, PMID: 33095478

