# Supplementary material for: Essential Roles of the Linker Sequence Between Tetratricopeptide Repeat Motifs of Ethylene Overproduction 1 in Ethylene Biosynthesis
Source: Front Plant Sci. 2021 Apr 15;12:657300. doi: 10.3389/fpls.2021.657300 (PMC8081955; doi:10.3389/fpls.2021.657300)
Supplement: Supplementary file 1 [file Data_Sheet_1.docx]

**Supplemental Information**


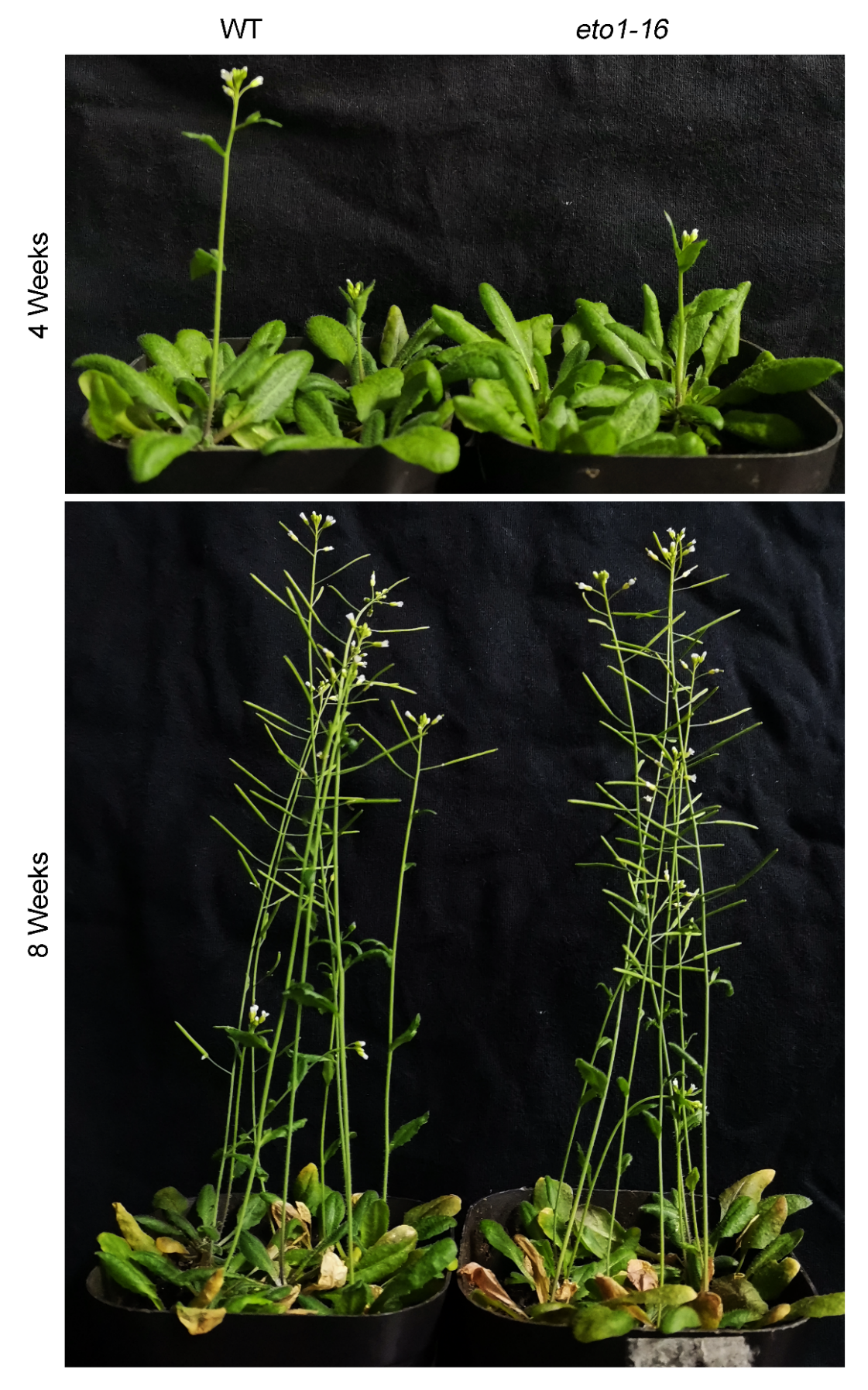


**Supplementary Figure 1** Growth phenotype of 4-week-old and 8-week-old wild type and *eto1-16* plants.


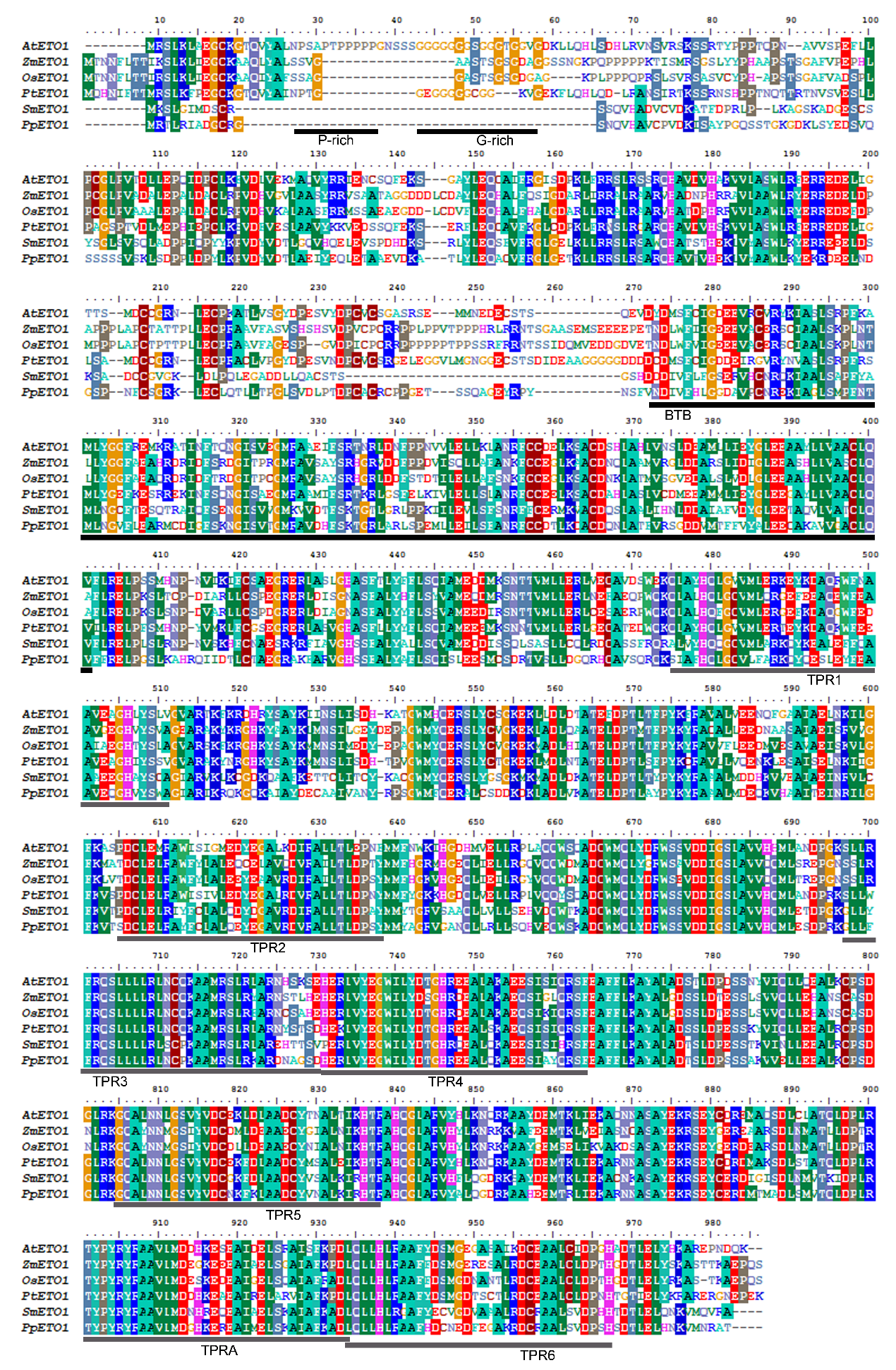


**Supplementary Figure 2** Full length sequence alignment of ETO1 in plants.


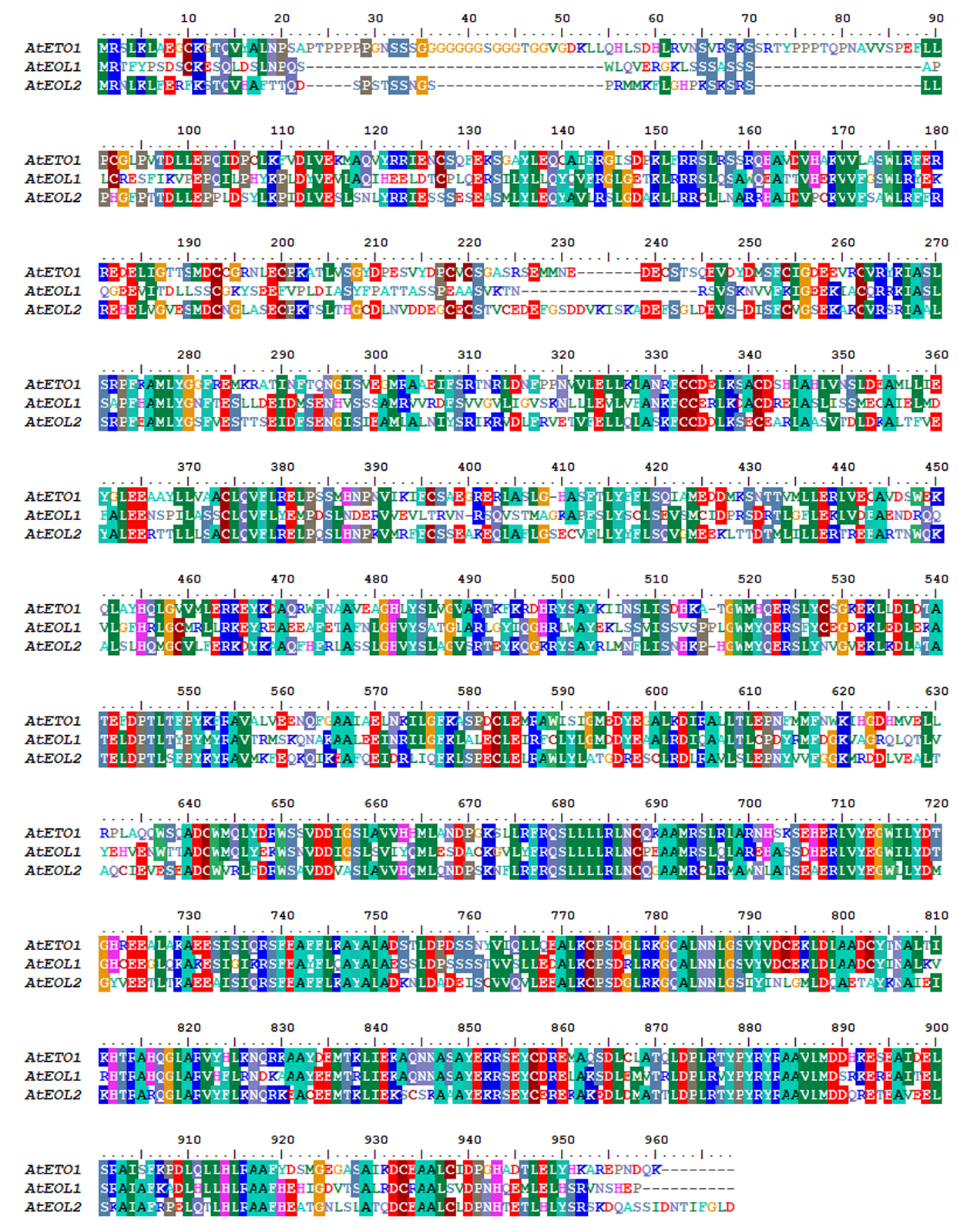


**Supplementary Figure 3** Full length sequence alignment of ETO1, EOL1 and EOL2 in *Arabidopsis*.


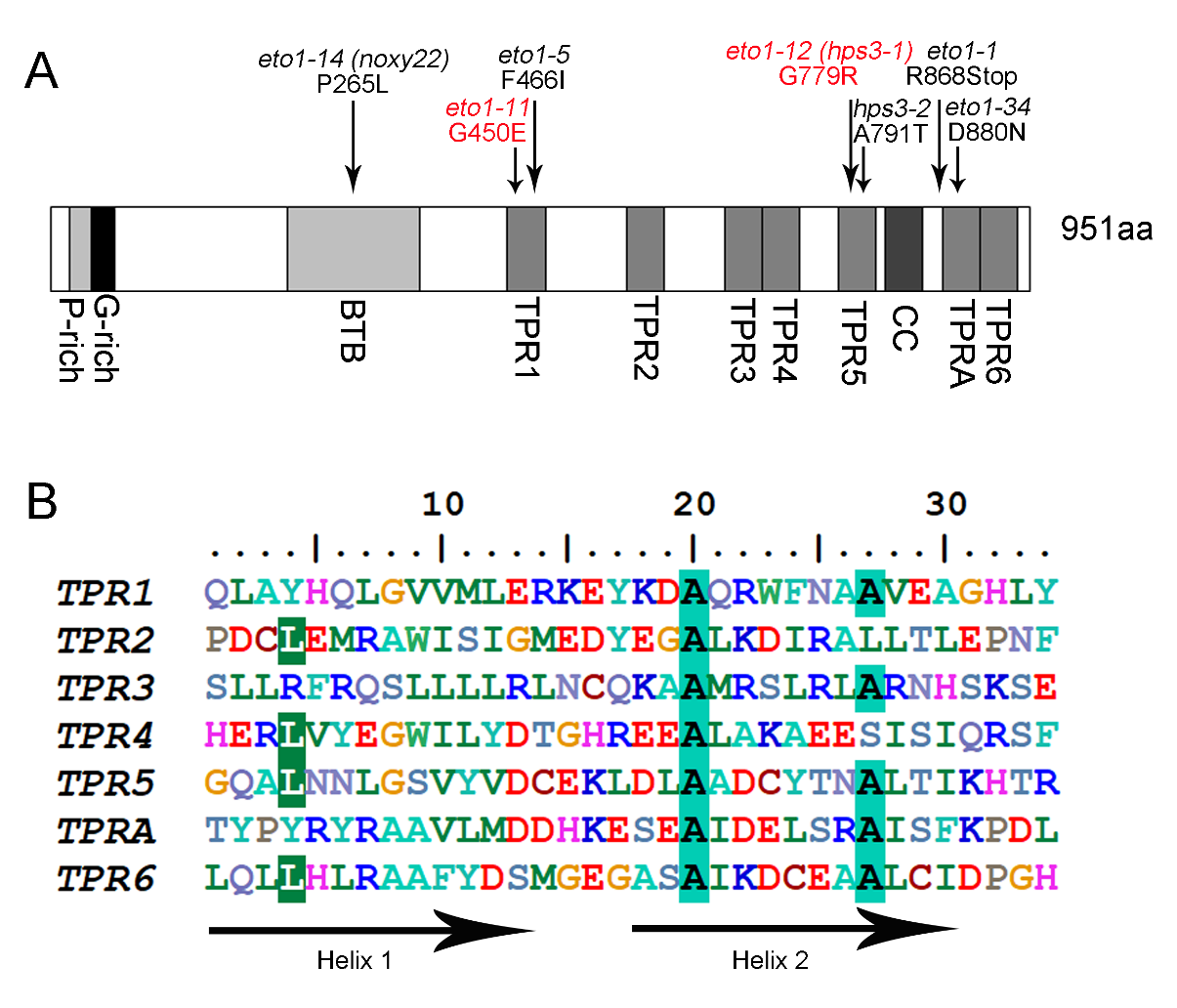


**Supplementary Figure 4** Missense *eto1* mutants and the TPR repeats in ETO1. A. Missense *eto1* mutants in the TPR domain and BTB domain, respectively. B. The helix motifs in TPR repeats of ETO1.


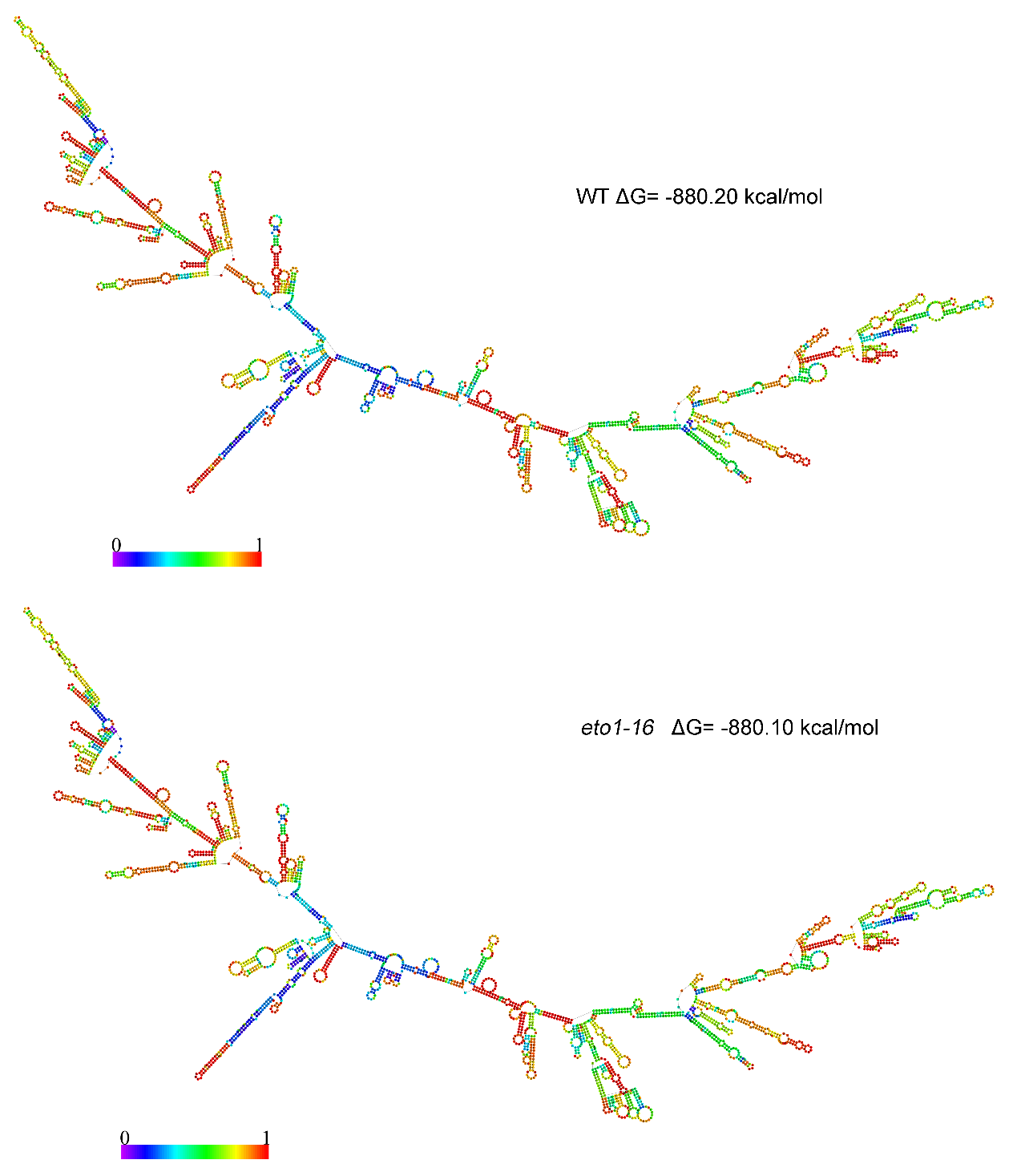


**Supplementary Figure 5** Predictive RNA secondary structure of WT and *eto1-16*.


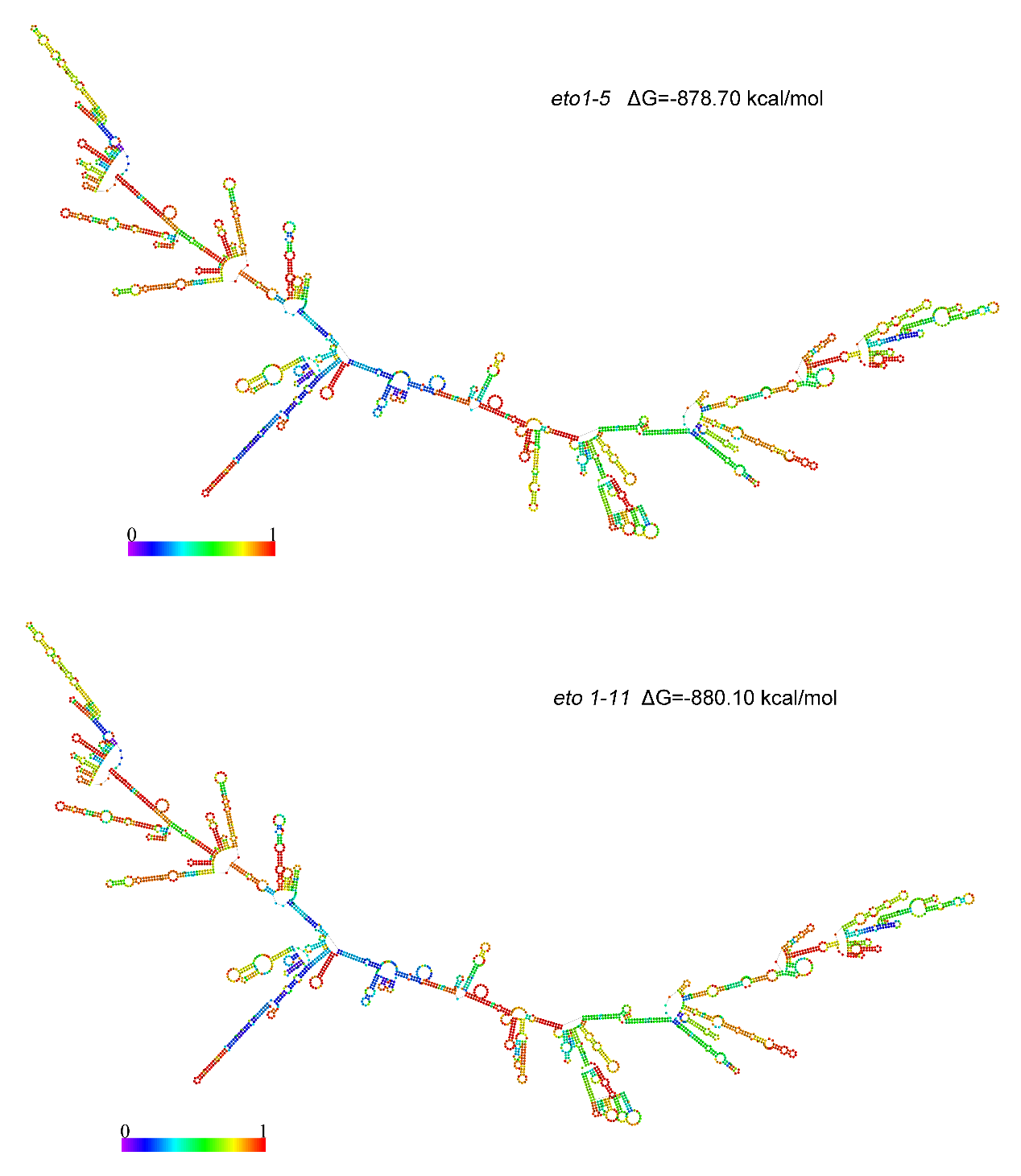


**Supplementary Figure 6** Predictive RNA secondary structure of *eto1-5* and *eto1-11*.


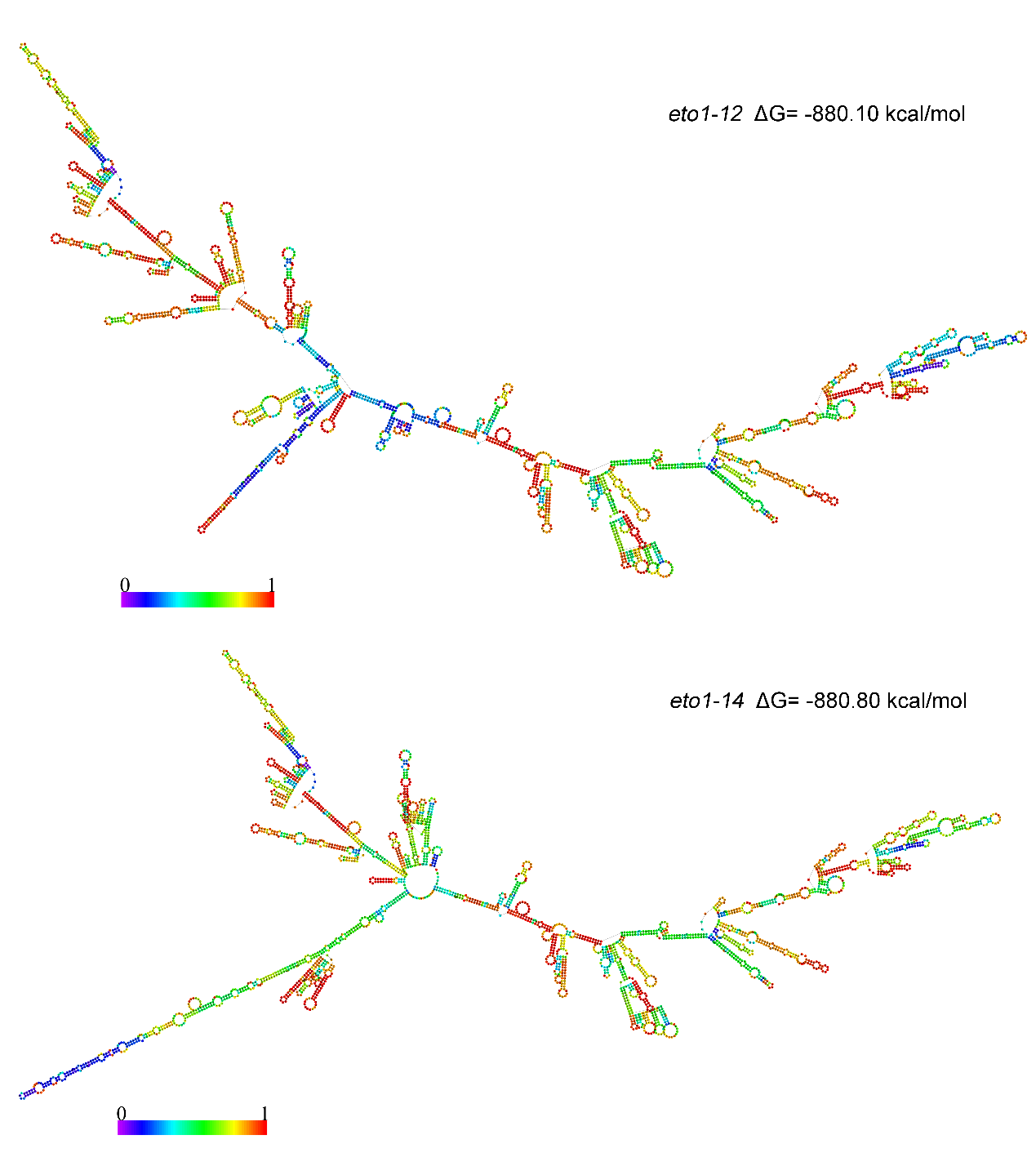


**Supplementary Figure 7** Predictive RNA secondary structure of *eto1-12* and *eto1-14*.


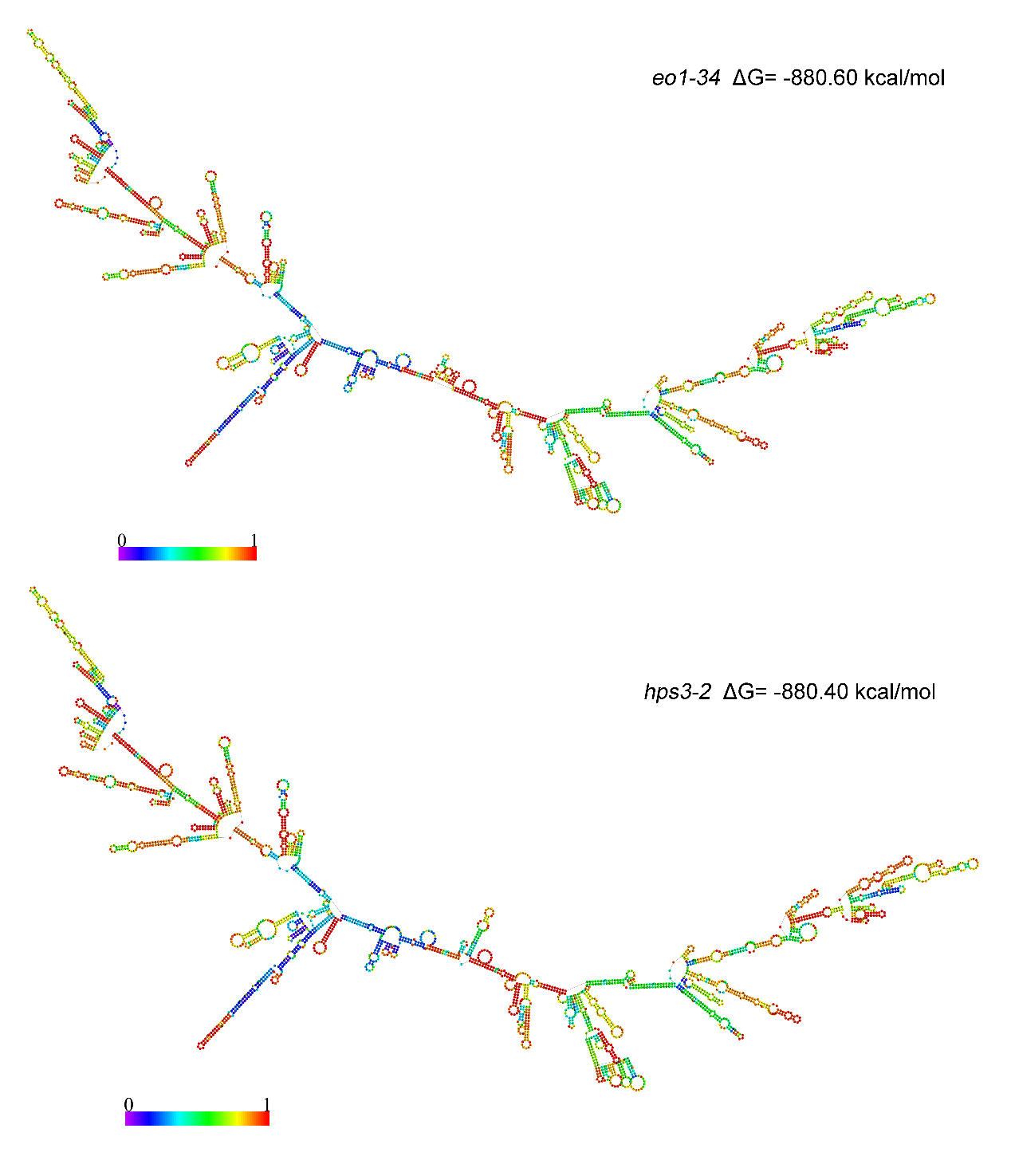


**Supplementary Figure 8** Predictive RNA secondary structure of *eto1-34* and *hsp3-2*.


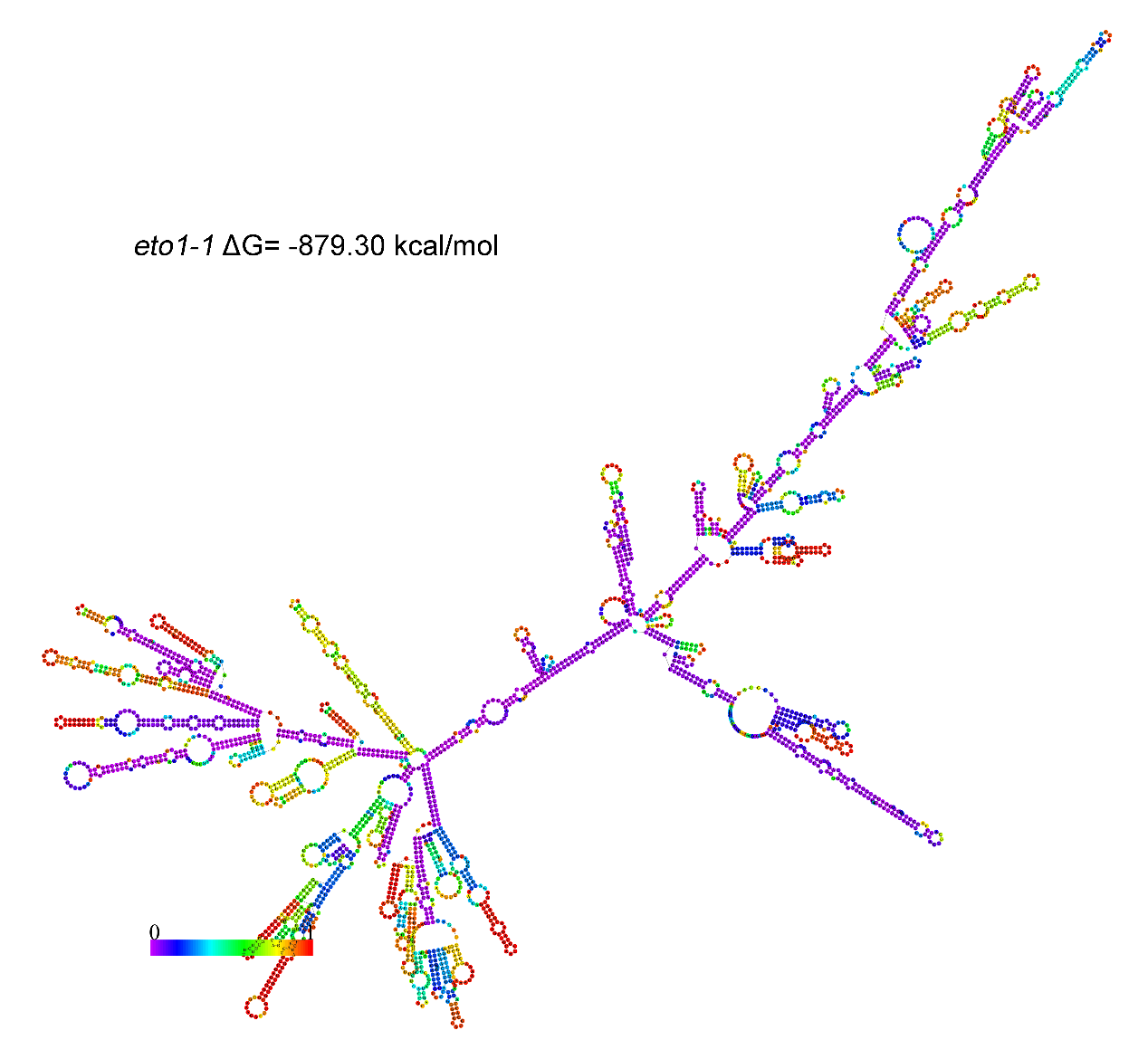


**Supplementary Figure 9** Predictive RNA secondary structure of *eto1-1*.


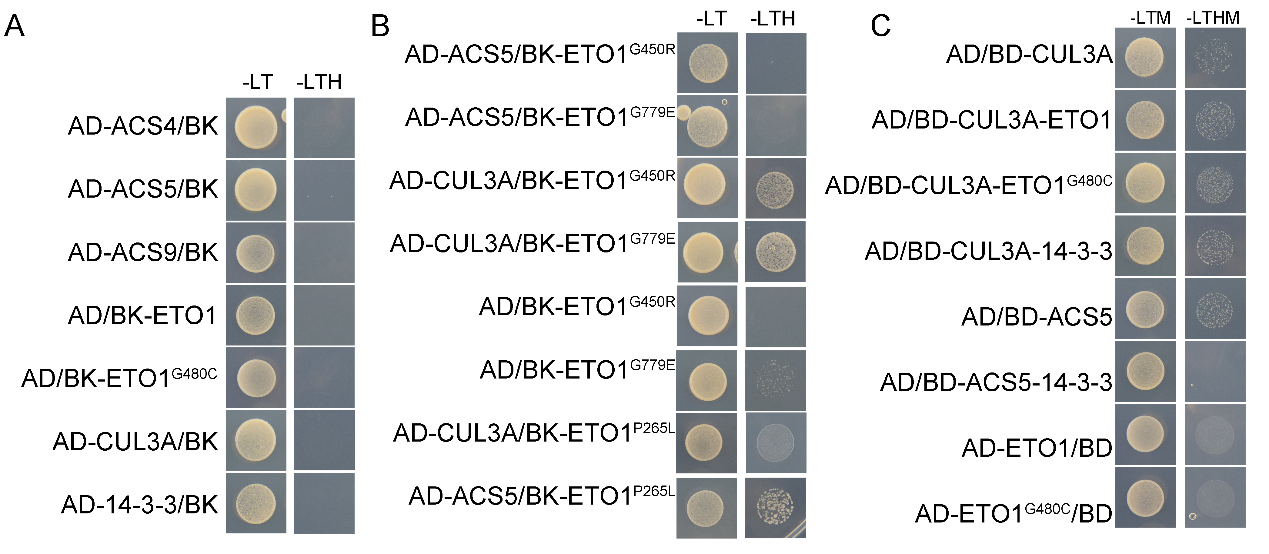


**Supplementary Figure 10** Negative controls in yeast hybrid assays and Y2H results for *eto1-11*, *eto1-12* and *eto1-14* mutants. A. Control groups in yeast two hybrid assay. B. Y2H results of ETO1 with ACS5 and CUL3A for *eto1-11*, *eto1-12* and *eto1-14* mutants. C. Control groups in Y3H assays.


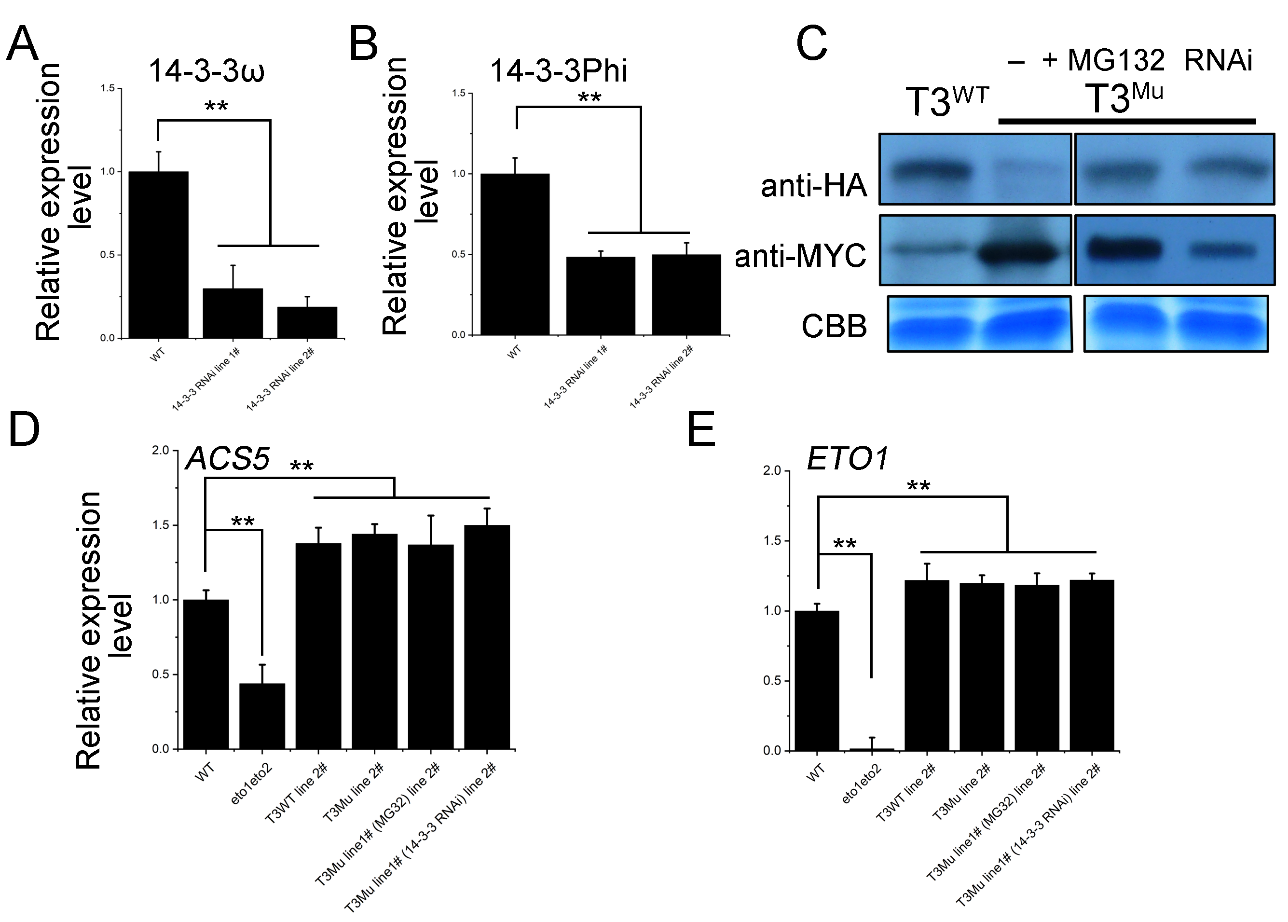


**Supplementary Figure 11** qRT-PCR analysis of *14-3-3*, *ACS5* and *ETO1* levels and western blot analysis of MYC-ACS5, HA-ETO1, and HA-ETO1^G480C^. A. The expression level of *14-3-3ω* in WT and *14-3-3ω* RNAi plants. B. the expression level of *14-3-3Phi* in WT and *14-3-3ω* RNAi plants. C. Western blot analysis of MYC-ACS5, HA-ETO1, and HA-ETO1^G480C^ in T3^WT^, T3^Mu^, T3^Mu^ (MG132) and T3^Mu^ (14-3-3 RNAi) plants. D. The expression level of *ACS5* in WT, *eto1eto2*, T3^WT^, T3^Mu^, T3^Mu^ (MG132) and T3^Mu^ (14-3-3 RNAi) plants. E. The expression level of *ETO1* in WT, *eto1eto2*, T3^WT^, T3^Mu^, T3^Mu^ (MG132) and T3^Mu^ (14-3-3 RNAi) plants. Error bars represent standard errors. Student’s t-test (**p < 0.01).


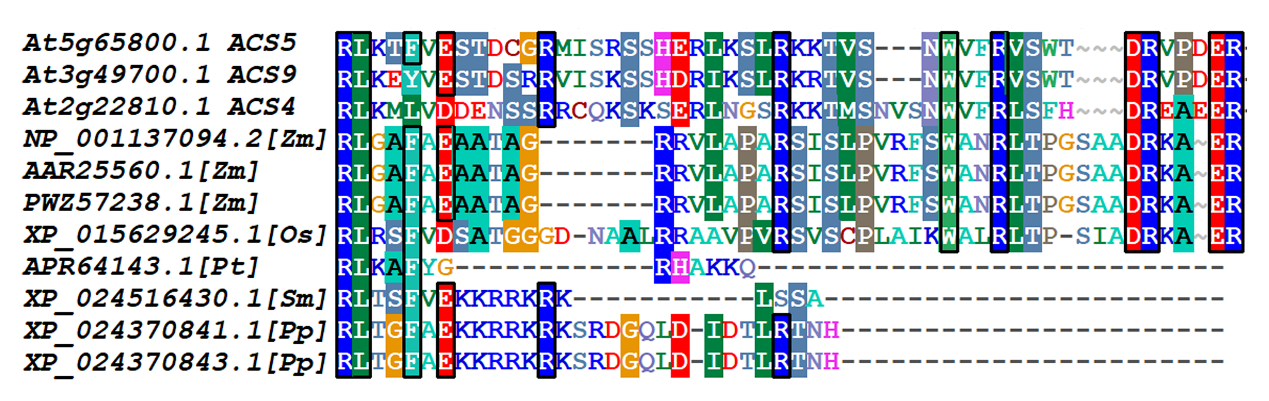


**Supplementary Figure 12** Sequence alignment of the C-termini of ACS proteins in different species.


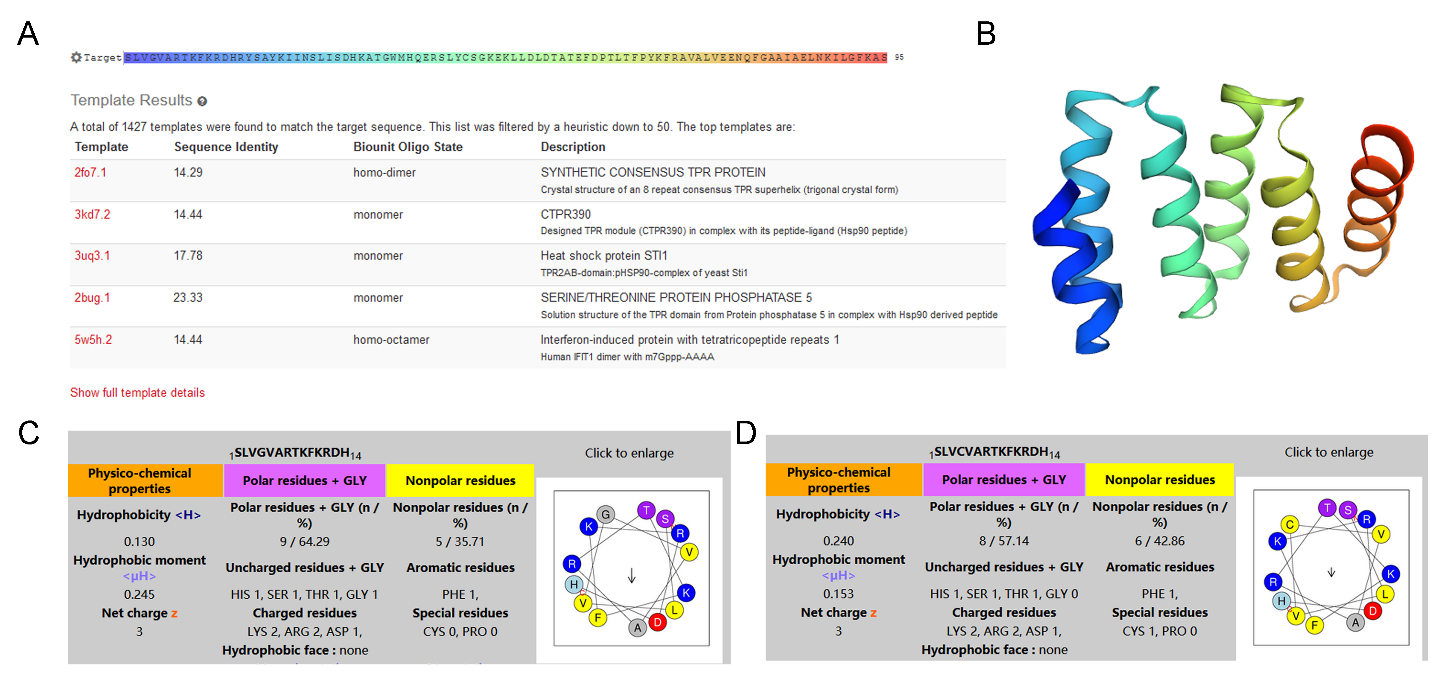


**Supplementary Figure 13** Similarity search and effects of the G480C on the potential helix formation of the linker sequence. A. Protein structure homology-modelling results for the linker sequence. B. Helix structure of 2bug.1 in PDB. C. Helix prediction of original linker sequence. D. The helix prediction data for the linker sequence with G480C mutation showed obvious discrepancies in Hydrophobicity and Hydrophobic moment values.


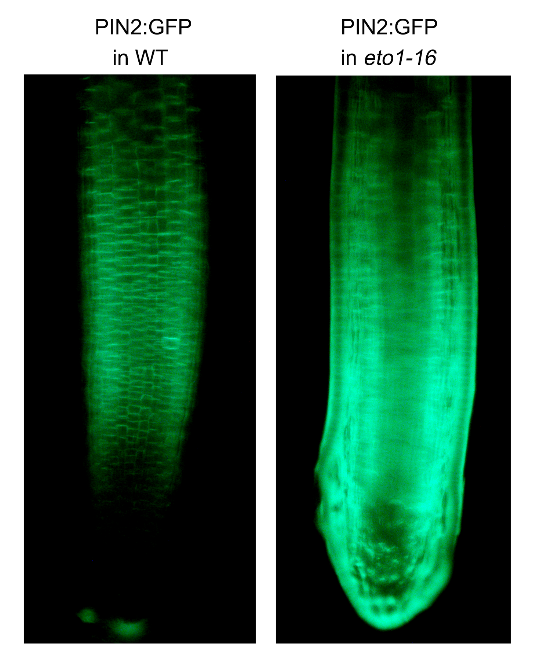


**Supplementary Figure 14** PIN2:GFP expression pattern in wild type and *eto1-16* plants.

**Supplementary Table 1** Primer sequences used in this study.

| **Primer name** | **Sequence** | **Usage** |
| --- | --- | --- |
| ETO1-1 | 5'-CCTGGATCCGATGGAATCCACTCTTCTTCAAG-3' | Complementary assay |
| ETO1-2 | 5'-CCTACGCGTGAAGCAATAATACATCTAGAAATAGGACAG-3' | Complementary assay |
| ETO1-aF | 5'-ATGAAATCTAACACAACTGTGATG-3' | RT-PCR |
| ETO1-aR | 5'-CAATTGAGCCGTAGGAGAAG-3' | RT-PCR |
| ETO1-bF | 5'-GTAAGGGACAAGCATTGAACAAC-3' | RT-PCR |
| ETO1-bR | 5'-CGTCCATAAGAACTGCGGCTC-3' | RT-PCR |
| ETO1-qF | 5'-GTAAGGGACAAGCATTGAACAAC-3' | qPCR |
| ETO1-qR | 5'-CAAGACCTTGGTGAGCTCGTG-3' | qPCR |
| Actin7-F | 5'-GATGCTTACGTTGGTGATGA-3' | RT-PCR |
| Actin7-R | 5'-CTGACTCATCGTACTCACT-3' | RT-PCR |
| Actin7-qF | 5'-TCCAACAGATGTGGATTTCAA-3' | qPCR |
| Actin7-qR | 5'-AACAAACTCACCACCACGAA-3' | qPCR |
| ACS4-1 | 5'-CCTCATATGGTTCAATTGTCAAGAAAAGC-3' | Y2H in AD |
| ACS4-2 | 5'-CCTCTCGAGCTATCGTTCCTCAGCCTCAC-3' | Y2H in AD |
| ACS5-1 | 5'-CCACATATGAAACAGCTTTCGACAAAAG-3' | Y2H in AD |
| ACS5-2 | 5'-CCTGGATCCTCATCGTTCATCAGGTACACGATC-3' | Y2H in AD |
| ACS9-1 | 5'-CCACATATGAAACAACTGTCGAGAAAAG-3' | Y2H in AD |
| ACS9-2 | 5'-CCTGAGCTCTCATCGTTCATCAGGATCACGGTC-3' | Y2H in AD |
| CUL3A-1 | 5'-CCTCATATGAGTAATCAGAAGAAGAGGAATTTTCAG-3' | Y2H in AD |
| CUL3A-2 | 5'-CCTCTCGAGTTAGGCTAGATAGCGGTAAAGTTTCC-3' | Y2H in AD |
| 14-3-3F | 5'-CCTCATATGGCGTCTGGGCGTGAAG-3' | Y2H in AD |
| 14-3-3R | 5'-CCTCTCGAGTCACTGCTGTTCCTCGGTCG-3' | Y2H in AD |
| ETO1-3 | 5'-CCTGAATTCATGCGAAGTTTGAAGCTTGC-3' | Y2H in BK |
| ETO1-4 | 5'-CCTGGATCCCTACTTTTGGTCGTTTGGTTCACG-3' | Y2H in BK |
| ACS5-3 | 5'-GTTTCGCTAATATGAGCGAGG-3' | qPCR |
| ACS5-4 | 5'-CTTAGGGAGATGAATAATTTCATCG-3' | qPCR |
| CUL3A-3 | 5'-CCTGAATTCATGAGTAATCAGAAGAAGAGGAATTTTCAG-3' | Y3H |
| CUL3A-4 | 5'-CCTAGATCTTTAGGCTAGATAGCGGTAAAGTTTCC-3' | Y3H |
| ACS5-5 | 5'-CCACATATGAAACAGCTTTCGACAAAAG-3' | Y3H in pBridge BD |
| ACS5-6 | 5'-CCTGGATCCTCATCGTTCATCAGGTACACGATC-3' | Y3H in pBridge BD |
| CUL3A-3 | 5'-CCTGAATTCATGAGTAATCAGAAGAAGAGGAATTTTCAG-3' | Y3H in pBridge BD |
| CUL3A-4 | 5'-CCTTTAGGCTAGATAGCGGTAAAGTTTCC-3' | Y3H in pBridge BD |
| ETO1-15： | 5'-CCTGCGGCCGCATGCGAAGTTTGAAGCTTGC-3' | Y3H in pBridge MSII |
| ETO1-16 | 5'-CCTGGATCCCTACTTTTGGTCGTTTGGTTCACG-3' | Y3H in pBridge MSII |
| ETO1-3 | 5'-CCTGAATTCATGCGAAGTTTGAAGCTTGC-3' | Y3H in AD |
| ETO1-4 | 5'-CCTGGATCCCTACTTTTGGTCGTTTGGTTCACG-3' | Y3H in AD |
| 14-3-3F2 | 5'-CCTGCGGCCGCGCGTCTGGGCGTGAAG-3' | Y3H in pBridge MSII |
| 14-3-3R2 | 5'-CCTCGATCCTCACTGCTGTTCCTCGGTCG-3' | Y3H in pBridge MSII |
| ACS4-3 | 5'-CCTGGATCCATGGTTCAATTGTCAAGAAAAGC-3' | BiFC in 3302YN |
| ACS4-4 | 5'-CCTACGCGTCTATCGTTCCTCAGCCTCAC-3' | BiFC in 3302YN |
| ACS5-7 | 5'-CCAGGATCCATGAAACAGCTTTCGACAAAAG-3' | BiFC in 3302YN |
| ACS5-8 | 5'-CCTACGCGTTCATCGTTCATCAGGTACACGATC-3' | BiFC in 3302YN |
| ACS9-3 | 5'-CCAGGATCCATGAAACAACTGTCGAGAAAAG-3' | BiFC in 3302YN |
| ACS9-4 | 5'-CCTACGCGTTCATCGTTCATCAGGATCACGGTC-3' | BiFC in 3302YN |
| ETO1-5 | 5'-CCTGGATCCATGCGAAGTTTGAAGCTTGC-3' | BiFC in 3302YC |
| ETO1-6 | 5'-CCTACGCGTCTACTTTTGGTCGTTTGGTTCACG-3' | BiFC in 3302YC |
| ETO1-7 | 5'-TACCCATACGACGTACCAGATTACGCTATGCGAAGTTTGAAGCTTGC-3' | HA-ETO1 construction |
| ETO1-8 | 5'-TGTTGTGAAGAGATTATGCTGCATCTTCAAATCAAAAACAACC-3' | HA-ETO1 construction |
| ACS5-9 | 5'-CCTGGATCCCCTAGCTCCACTTGTCTCTG-3' | MYC-ACS5 construction |
| ACS5-10 | 5'-AGGAGCAGAAGCTGATCTCAGAGGAGGACCTGATGAAACAGCTTTCGACA AAAG-3' | MYC-ACS5 construction |
| ACS5-11 | 5'- TCTCTGTTTTTAAAGTCAAGAGATTTTGGTCGTAGAGAAGAC-3' | MYC-ACS5 construction |
| ACS5-12 | 5'-CCTCCATGGGCCACTGCAAATCCACATTCGGATTGAG-3' | MYC-ACS5 construction |
| 14-3-3RNAi-1: | 5'-CCTGGATCCACGTCACGGCGATCCGTGAATATAGG-3' | RNAi plants construction |
| 14-3-3RNAi-2 | 5'-CCTACGCGTGGAGCAAGCTCTGCATTAGCAATATC-3' | RNAi plants construction |
| 14-3-3RNAi-3 | 5'-CCTACGCGTTGTTCGGCGGCGTCTTTCCTC-3' | RNAi plants construction |
| 14-3-3RNAi-4 | 5'-GGATCCACGTCACGGCGATCCGTGAATATAGG-3' | RNAi plants construction |

**Supplementary Table 2** Frequency of mutants in F2 population of *eto1-16* crossed with Landsberg erecta.

| Cross (F2) | Number of plants | | | χ2a |
| --- | --- | --- | --- | --- |
|  | Mutants | WT | Total |  |
| *eto1-16**Ler | 54 | 181 | 235 | 0.410 |

^a^. The calculated χ2 value was based on the expected ratio of 1:3 for mutants to WT, assuming that *eto1-16* is a single recessive mutation (P > 0.05).
